# Engineering a Rhodopsin-Based Photo-Electrosynthetic System in Bacteria for CO_2_ Fixation

**DOI:** 10.1021/acssynbio.2c00397

**Published:** 2022-10-20

**Authors:** Paul A. Davison, Weiming Tu, Jiabao Xu, Simona Della Valle, Ian P. Thompson, C. Neil Hunter, Wei E. Huang

**Affiliations:** † Plants, Photosynthesis and Soil, School of Biosciences, University of Sheffield, SheffieldS10 2TN, United Kingdom; ‡ Department of Engineering Science, University of Oxford, OxfordOX1 3PJ, United Kingdom

**Keywords:** *Ralstonia eutropha*, β-carotene, proteorhodopsin, *Gloeobacter* rhodopsin, biosynthesis, synthetic biology, CO_2_ fixation, photoautotrophy

## Abstract

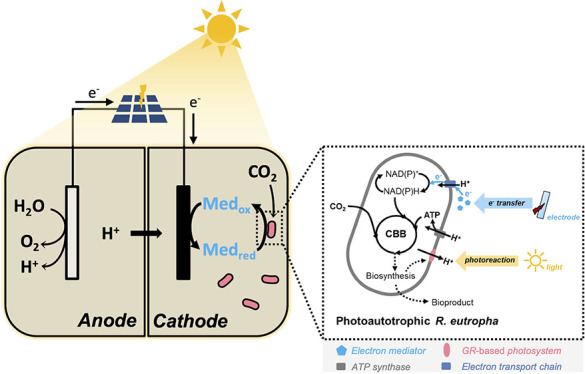

A key goal of synthetic biology is to engineer organisms that can use solar energy to convert CO_2_ to biomass, chemicals, and fuels. We engineered a light-dependent electron transfer chain by integrating rhodopsin and an electron donor to form a closed redox loop, which drives rhodopsin-dependent CO_2_ fixation. A light-driven proton pump comprising *Gloeobacter* rhodopsin (GR) and its cofactor retinal have been assembled in *Ralstonia eutropha* (*Cupriavidus necator*) H16. In the presence of light, this strain fixed inorganic carbon (or bicarbonate) leading to 20% growth enhancement, when formate was used as an electron donor. We found that an electrode from a solar panel can replace organic compounds to serve as the electron donor, mediated by the electron shuttle molecule riboflavin. In this new autotrophic and photo-electrosynthetic system, GR is augmented by an external photocell for reductive CO_2_ fixation. We demonstrated that this hybrid photo-electrosynthetic pathway can drive the engineered *R. eutropha* strain to grow using CO_2_ as the sole carbon source. In this system, a bioreactor with only two inputs, light and CO_2_, enables the *R. eutropha* strain to perform a rhodopsin-dependent autotrophic growth. Light energy alone, supplied by a solar panel, can drive the conversion of CO_2_ into biomass with a maximum electron transfer efficiency of 20%.

## Introduction

One vital challenge in the 21st century is the sustainable production of chemicals and fuels from CO_2_, using a biocatalyst and driven by a renewable energy source, for example from sunlight.^1,2^ Harnessing the biological fixation of CO_2_ for sequestering biomass is an ideal outcome in terms of mitigating rising levels of atmospheric CO_2_, even more so if useful products such as chemicals and fuels could be generated. Recent important advances in synthetic biology have demonstrated that *Escherichia coli* can be genetically engineered to grow on CO_2_ while reducing power and energy are obtained by oxidizing a supplied organic compound (e.g., pyruvate and formate).^3−5^ Other approaches harnessed the native CO_2_ fixation pathway of the chemolithotroph *Ralstonia eutropha* H16, coupled to an electrochemical supply of the required reducing power.^6,7^ Solar-powered electrochemical systems producing H_2_ or formate combined with *Ralstonia eutropha* H16 can drive artificial photosynthetic processes for carbon fixation into biomass and biofuels.^8^

The engineering of photoautotrophic growth into a synthetic biology chassis would represent the next level of metabolic engineering, in terms of using synthetic biology for sustainable production of biomass, chemicals, and fuels. Engineering photoautotrophy would require exploitation of one of the light-driven systems that have naturally evolved, which are based on either chlorophyll or rhodopsins.^9−11^ Chlorophyll-based photosynthesis requires a large and relatively complex network of components for light harvesting, charge separation, and electron and proton transfers, for driving CO_2_ fixation in cells. In contrast, rhodopsin-based utilization of light is simpler, involving only one membrane protein^12^ that usually comprises seven transmembrane α-helices. Microbial rhodopsins are widespread among microbial inhabitants of sunny environments such as the upper ocean,^13^ and their functions have been dissected by heterologous production in *E. coli*.^10,14,15^ These studies demonstrated that retinal-binding proteorhodopsin (PR) generates a proton gradient that can be used for the production of biologically available energy in the form of ATP.^14,16^ Although microbial rhodopsins have been reported as major contributors to the solar energy capture in the sea,^11^ there is no report of rhodopsin-driven autotrophic growth in microbes, likely due to the lack of electron donors required for the reductive assimilation of CO_2_. In contrast, chlorophyll-based photoautotrophic systems obtain electron donors from light-driven water splitting by photosystem II, and photosystem I uses light to generate a proton-motive force for ATP generation. Inspired by chlorophyll-based photoautotrophy,^17^ we hypothesized that a closed redox loop can be constructed by integrating rhodopsin with an electron donor. If the electron donor can be supplied by an electrode powered by a solar panel, a rhodopsin-based photo-electrosynthetic system could drive the autotrophic growth of bacteria using CO_2_ as the sole carbon source and with light as the only energy input.

**Figure 1:**
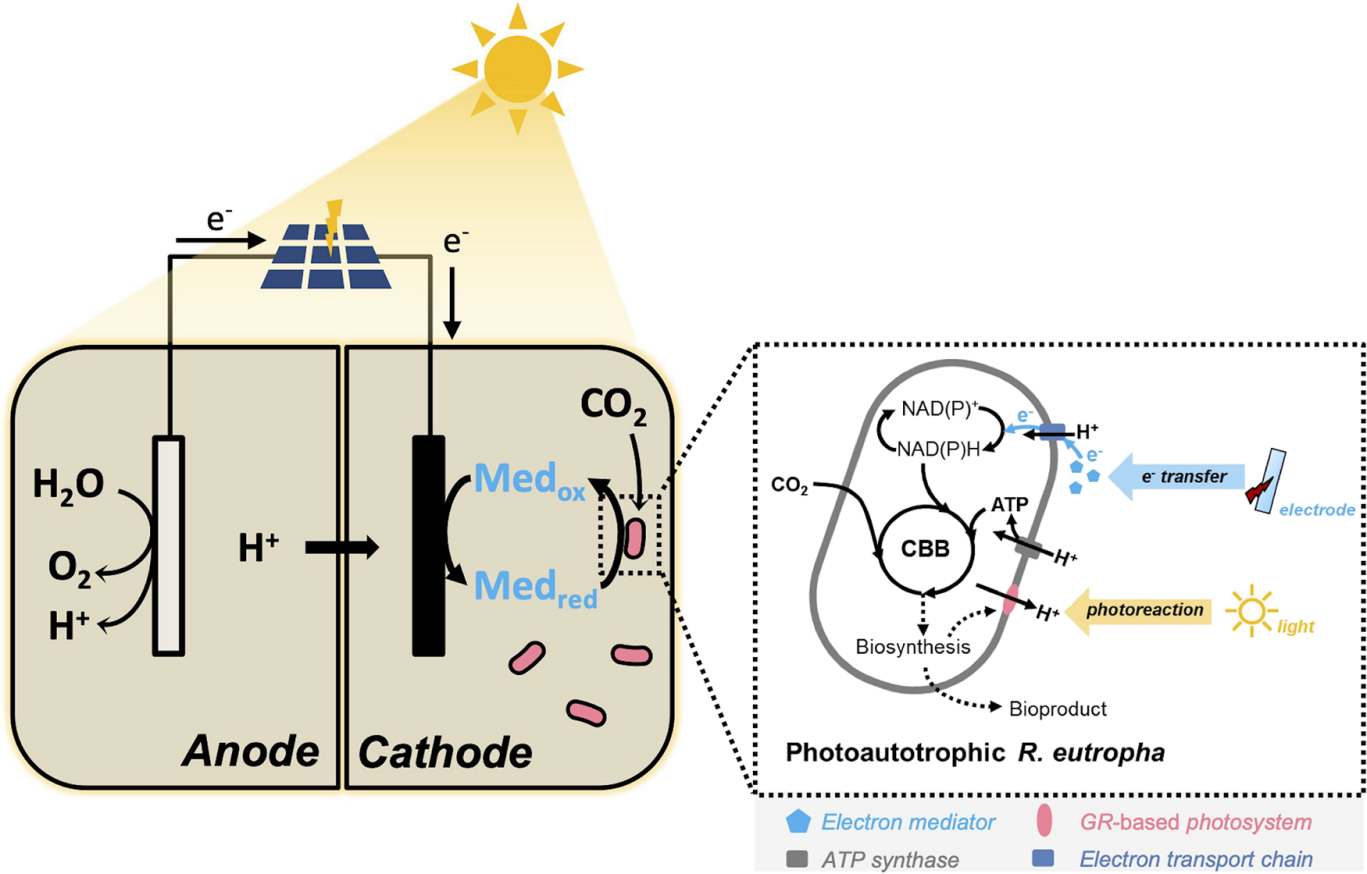
A rhodopsin-based photoautotrophic system is able to fix CO_2_. Light can be used to activate rhodopsin and generate electricity. Light-activated rhodopsin pumps protons and, when coupled with ATP synthase, generates ATP. An electrode, mediated by riboflavin, can serve as an electron donor to supply electrons. The closed redox loop drives CO_2_ fixation.

To test this hypothesis, we engineered a light-powered electromicrobial system for CO_2_ fixation (Figure 1). The chosen bacterium, *Ralstonia eutropha* (*Cupriavidus necator*) H16, lacks the capacity to use light as an energy source,^18,19^ and its native CO_2_ fixation pathway is encoded on two operons of the Calvin–Benson–Bassham (CBB) cycle, one on chromosome 2 and the other on its pHG1 megaplasmid.^20^ In this study, *R. eutropha* H16 was engineered to synthesize rhodopsin that harvests light energy to generate ATP, while an electron-carrying molecule is required for the electron transfer from an electrode to cells. *R. eutropha* H16 commonly utilizes H_2_ generated from water splitting to drive CO_2_ reduction.^21^ Nevertheless, the generation of H_2_ (or formate) is generally catalyzed by an abiotic electrode and the metallic catalysts usually dissolve into the medium and produce toxic compounds to inhibit the biosynthesis or growth of *R. eutropha* H16. In this study, the biocompatible and less toxic molecule riboflavin was introduced as an electron shuttle to mediate the electron transfer from an electrode. This bioenergetic system has only two inputs, light and CO_2_. The bioenergy for the *R. eutropha* H16 growth is supplied by installing *Gloeobacter violaeus* rhodopsin (GR), augmented by an external photocell that serves as the electron donor (Figure 1). This engineered bacterium performs a hybrid form of photoautotrophy, which uses light to convert CO_2_ into biomass.

## Results

### Biosynthesis of β-Carotene in *R. eutropha*

**Figure 2:**
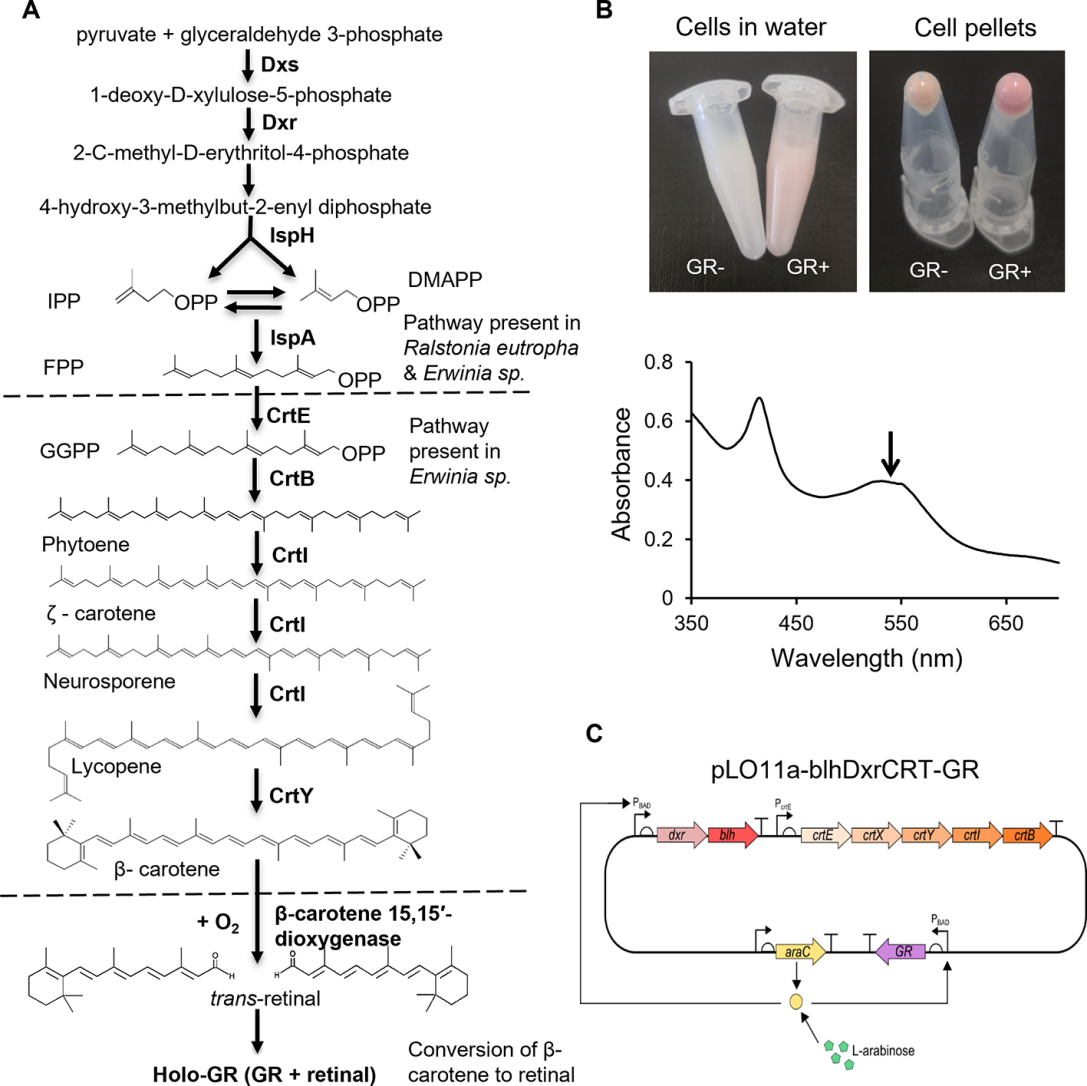
(A) Pathway showing the synthesis of β-carotene from pyruvate and glyceraldehyde 3-phosphate and its conversion to retinal by β-carotene 15,15′-dioxygenase (product key: IPP, isopentenyl diphosphate; DMAPP, dimethylallyl pyrophosphate; FPP, farnesyl diphosphate; GGPP, geranylgeranyl diphosphate). The parts of the pathway common to both *Ralstonia eutropha* and *Erwinia* sp. and unique to *Erwinia* sp. are indicated. The enzyme key is as follows: Dxs, 1-deoxy-d-xylulose-5-phosphate synthase; Dxr, 1-deoxy-d-xylulose 5-phosphate reductoisomerase; IspH, 4-hydroxy-3-methylbut-2-enyl diphosphate reductase; crtE, geranylgeranyl diphosphate synthase; crtB, phytoene synthase; crtI, phytoene desaturase; crtY, lycopene cyclase. (B) GR expression in *R. eutropha* H16 causes a distinct pink coloration. Absorbance scan of isolated membranes from the induced H16 GR cells shows absorption at 540 nm (arrowed), a light wavelength that is not absorbed by chlorophyll. (C) The gene-circuit design shows the arrangement of the pLO11a-blhDxrCRT-GR construct. This contains the crt operon (*crtEXYIB*) plus an 879 bp upstream region containing the *crtE* endogenous promoter (P_crtE_) and the *dxr*, *blh*, and *GR* genes (with upstream ribosome binding site shown as semi-circles) under the control of the arabinose inducible P_BAD_ promoter.

Biosynthesis of carotenoids requires a supply of the geranylgeranyl diphosphate (GGPP) precursor, followed by phytoene desaturation and subsequent modifications such as cyclization or introduction of keto groups.^22^ The carotenoid β-carotene is the precursor of retinal, which is the essential cofactor for the light-driven proton pump rhodopsin. The entire *crtEXYIB* operon for β-carotene synthesis, including the *crtE* promoter region (*crt* operon), from the pORANGE plasmid^23^ was cloned into the pLO11a plasmid to make pCRT (Table 1 and Suppl Figure S1A). The resulting production of β-carotene by *R. eutropha* H16 can be enhanced by overexpression of the *dxr* gene in pDxrCRT (Table 1 and Suppl Figure S1B). A biosynthetic pathway for β-carotene synthesis in *R. eutropha* H16 is shown in Figure 2A. Detailed information about the production of β-carotene in *R. eutropha* H16 (strain H16 pDxrCRT) is given in the Supplementary Information and Figure S2.

**Table 1:** Strains and Plasmids Used in This Study

**strains**	**comments**
*E. coli* JM109	Commercially obtained (Promega, UK)
*Ralstonia eutropha* H16 (ATCC 17699)	Wild-type strain
*Ralstonia eutropha* H16 Δ*pha* (RHM5)	Deletion of phaCAB operon encoding genes required for conversion of acetyl-CoA to polyhydroxybutyrate (PHB) (gift from Min-Kyu Oh, Korea University, S. Korea).
**plasmids**	**comments**
pLO11a (Tc^r^, RK2 *ori*, Mob^+^)	Expression vector for use in *R. eutropha* with the P_BAD_ promoter and downstream cloning sites (gift from Oliver Lenz, Technische Universität Berlin, Germany).
pLO11a-CrtYI	pLO11a containing *crtY* and *crtI* from *Erwinia herbicola*
pLO11a-CRT	pLO11a containing constitutive *crtEXYIB* operon from *Erwinia uredovora*
pLO11a-Dxr	pLO11a containing *dxr* from *R. eutropha*
pLO11a-DxrCRT	pLO11a containing *dxr* from *R. eutropha* downstream of P_BAD_ promoter and upstream of *crtEXYIB* operon from *Erwinia uredovora*
pLO11a-GR	pLO11a containing the gene for GR rhodopsin from *Gloeobacter violaceus* PCC7421
pLO11a-blh	pLO11a containing *blh* from the uncultured marine bacterium 66A03 (GenBank: DQ065755.1) codon optimized for *R. eutropha*
pLO11a-Dxr-blh	pLO11a containing *dxr* and *blh* genes separated by a ribosome binding site
pLO11a-blhDxrCRT	pLO11a containing *dxr* and *blh* genes separated by a ribosome binding site downstream of the P_BAD_ promoter and upstream of *crtEXYIB* operon from *Erwinia uredovora*
pLO11a-blhDxrCRT-GR	pLO11a-blhDxrCRT containing the *GR* gene downstream with its own P_BAD_ promoter

### Biosynthesis of *Gloeobacter* Rhodopsins in *R. eutropha*

GR is a rhodopsin from the cyanobacterium *Gloeobacter violaceus* PCC7421 (GenBank: BAC88139).^24,25^ Transfer of the plasmid pLO11a–GR into *R. eutropha* H16 resulted in a distinct pink color after growth to log phase and overnight induction in the presence of 0.1% (w/v) l-arabinose and exogenous 5 μg mL^–1^ of retinal (Figure 2B). To examine the GR–retinal complex present in the cytoplasmic membrane, a cell pellet from a 500 mL arabinose and retinal-supplemented culture of *R. eutropha* H16 containing pLO11a-GR was disrupted using a French press and the membrane fraction purified on a sucrose density gradient. The fractionated cell extract from arabinose-induced cells generated a diffuse, highly colored membrane band in the middle of the gradient. An absorbance spectrum (Figure 2B) recorded on a sample harvested from the middle of this band revealed a peak at around 540 nm (arrowed), which is consistent with the published value for the GR–retinal complex.^26^

### Construction of a Gene Cluster for GR–Retinal Complex Biosynthesis in *R. eutropha*

Following the construction of two *R. eutropha* strains that separately produce β-carotene (H16 pDxrCRT) and GR (H16 GR), these new attributes were combined within a single strain to enable the assembly of a functional rhodopsin that confers the capacity for ATP synthesis in the light. The *blh* gene from the uncultured marine bacterium 66A03 encodes the β-carotene 15, 15′-dioxygenase enzyme (EC:1.13.11.63), which cleaves one molecule of β-carotene into two molecules of retinal (Figure 2A) in the presence of oxygen.^27^ Although the H16 pDxrCRT strain successfully makes β-carotene, the conversion of this into retinal is a potentially rate-limiting step so the *blh* gene was codon optimized for *R. eutropha* to ensure the best chance of expression and cloned into pDxrCRT to create pLO11a-blhDxrCRT (Table 1), an expression vector for retinal production. The *GR* gene, under the control of a separate P_BAD_ promoter, was then inserted into this vector to create pLO11a-blhDxrCRT-GR, for the production of a GR holoprotein (Figure 2C and Table 1). This construct was transferred by conjugation into *R. eutropha* H16 to create *R. eutropha* H16 blhDxrCRT-GR (henceforth *R. eutropha*-GR), which acquired a pink color in the presence of 0.1% arabinose inducer, indicating the assembly of a GR–retinal holocomplex. Solvent extraction of the pellets followed by HPLC analysis showed that the uninduced sample contained no β-carotene (Suppl Figure S3A, broken line) or retinal (Suppl Figure S3B, broken line), but upon induction, a small amount of β-carotene (Suppl Figure S3A, solid line) and a larger amount of retinal, appearing as an elution peak at approximately 8 min under these running conditions, could be detected (Suppl Figure S3B, solid line). This peak had an absorbance spectrum identical to that seen for all *trans*-retinal with an absorbance maximum at 380 nm (Suppl Figure S3C, solid line). Following induction, two further peaks appeared at 8.9 and 9.2 min (Suppl Figure S3B labeled 1 and 2, respectively) with absorbance spectra (Suppl Figure S3C, broken lines) similar to all *trans*-retinal but with shifted absorbance maxima of 386 nm (peak 1) and 395 nm (peak 2). It is not known if these are isomers or derivatives of *trans*-retinal, although the expression of mouse β-carotene 15, 15′-dioxygenase in a β-carotene-expressing *E.coli* strain also resulted in the appearance of extra peaks in addition to that seen for retinal.^28^ Raman micro-spectroscopy was used to examine GR–retinal complexes in *R. eutropha* H16 and *E. coli* JM109. Single-cell Raman spectra (SCRS) of cells synthesizing GR display a unique band at 1530 cm^–1^ (Suppl Figure S4), which is a characteristic biomarker for the retinal–GR holoenzyme, similar to that previously reported for the retinal–PR holoenzyme.^29^

### GR Expression in *R. eutropha* H16 Significantly Enhances Cell Growth

**Figure 3:**
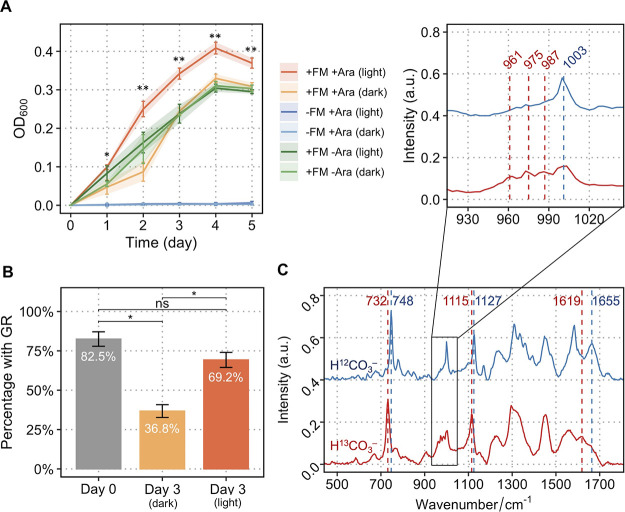
(A) Growth curves, represented by OD_600_, of *R. eutropha* H16 with pLO11a-blhDxrCRT-GR (*R. eutropha*-GR) grown under micro-aerobic conditions in minimal medium with and without 80 mM formate, with and without light, and with and without induction of GR expression. Independent *t* tests were performed: *p < 0.05, **p < 0.01. Data show means ± SD, *n* = 3. Legend key: FM: formate and Ara: arabinose. (B) Bar chart showing the percentage of the *R. eutropha*-GR cell population grown in the presence of formate expressing GR at day 0 and after 3 days in the light and dark. Independent *t* tests were performed: *p < 0.05. Data show means ± SD, *n* = 3. (C) SCRS of cells of *R. eutropha*-GR grown under light in either ^12^C-bicarbonate (blue) or ^13^C-bicarbonate (red). Cells grown in ^13^C-bicarbonate exhibited isotopic Raman shifts from 1655 to 1619 cm^–1^ (amide I of proteins), 1127 to 1115 cm^–1^ (cytochrome c), 748 to 732 cm^–1^ (cytochrome c), and 1002 to 987, 975, and 961 cm^–1^ (phenylalanine).

Initially, formate was used as the organic electron donor to investigate the light-dependent growth of the engineered *R. eutropha*-GR strain. Figure 3A shows that when arabinose was added as the inducer under micro-aerobic conditions, the *R. eutropha*-GR strain illuminated by white LED light showed an enhanced growth with the biomass increased up to 24% compared to that seen in the dark or in uninduced or dark-grown cells. Given the likely consumption of formate in the earlier stages of this experiment,^30^ the final increase in biomass at the later time points is ascribed to the light-driven turnover of GR and the subsequent production of ATP and NADPH for CO_2_ fixation. The micro-aerobic growth rate of *R. eutropha*-GR has increased to 0.022 h^–1^ in the light, compared to 0.015 h^–1^ in the dark. The light-enhanced growth was maintained over the 5-day time course of the experiment, indicating a significant light-dependent increase in biomass of the *R. eutropha*-GR strain. Control strains grown in the presence of arabinose alone (no formate) did not show any growth (Figure 3A), ruling out the possibility that the inducer can be used as a carbon source. *R. eutropha* H16 with no GR expression (containing pLO11a-blhDxrCRT-GR but no arabinose induction) showed no detectable growth difference between light and dark conditions (Figure 3A). Thus, this is evidence that holo-GR produced in *R. eutropha*-GR can capture light energy to increase cell growth in the presence of formate. The light energy harvested by GR significantly enhanced polyhydroxybutyrate (PHB) accumulation in *R. eutropha*-GR when formate was used as the sole carbon source (Suppl Figure S5). This also confirms that energy from light-driven proton pumping by GR stimulates biosynthesis.

Raman micro-spectroscopy was employed to examine cells containing GR. After the growth of *R. eutropha*-GR in minimal medium (MM) with formate in the dark or light for 3 days, the position of the 1530 cm^–1^ Raman band, characteristic for GR, remained the same (Suppl Figure S6). We further measured SCRS of the bacterial population (200 or 400 single cells) and calculated the percentage of the sub-population expressing GR, using the GR characteristic Raman band at 1530 cm^–1^ (Suppl Figure S4, Table S1 and Figure 3B). At the outset, 83% of the *R. eutropha* H16 population synthesized holo-GR (Figure 3B); this proportion declined only to 69% by day 3 under micro-aerobic conditions in the light, in the absence of antibiotic selection pressure, while it dropped to 37% in the dark (Table S1 and Figure 3B). This indicates that *R. eutropha*-GR in the light had a growth advantage over the cells without GR, leading to an enrichment of those cells with GR. Micro-aerobic conditions were used in this study to reduce the risk of overproduction of reactive oxygen species (ROS).^31^ The Raman analysis at the single-cell level showed that the percentage of GR-containing cells under micro-aerobic conditions was higher than that seen under aerobic conditions (Table S1).

### CO_2_ Fixation by GR-Expressing *R. eutropha* H16

Stable isotope probing was used to examine CO_2_ fixation by the *R. eutropha*-GR strain. Formate (40 mM) and bicarbonate (40 mM) were used under three differing conditions: (1) ^12^C-formate + ^12^C-bicarbonate, (2) ^13^C-formate + ^12^C-bicarbonate, and (3) ^12^C-formate + ^13^C-bicarbonate. Under these three labeling conditions, illumination for 3 days improved growth by 18% (Suppl Figure S7A), 20% (Suppl Figure S7B) and 20% (Suppl Figure S7C), respectively. Incorporation of a ^13^C substrate was detected via shifted Raman bands in SCRS,^32^ as shown in Figure 3C for *R. eutropha*-GR grown in ^13^C-formate and ^13^C-bicarbonate, compared to ^12^C substrates. In particular, two Raman bands corresponding to cytochrome c^33−35^ at 748 (pyrrole breathing mode) and 1127 cm^–1^ (υ (CN) stretching vibrations) and a band arising from proteins at 1655 cm^–1^ (amide I) are shifted to 732, 1115, and 1619 cm^–1^, respectively (Figure 3C). The single band at 1003 cm^–1^ is characteristic of the phenyl ring of ^12^C-phenylalanine, which becomes three bands at 987, 975, and 961 cm^–1^ in the ^13^C amino acid (Figure 3C), in good agreement with the ^13^C Raman shifts observed in a previous work.^36^ Isotope labeling with ^13^C-bicarbonate (Figure 3C) confirms CO_2_ fixation by *R. eutropha*-GR and subsequent incorporation into biomass. Although *R. eutropha*-GR cells, in both light and dark conditions, show their ability to assimilate bicarbonate (Suppl Figure S8), *R. eutropha*-GR in the light can reach higher biomass than that in the dark (Figure 3A).

We further sought to compare the extent of ^13^C incorporation from ^13^C-bicarbonate and ^13^C-formate in the light (Suppl Figure S9). The Raman band for pyrrole ring breathing in cytochrome c, originally at 748 cm^–1^ (Suppl Figure S9), exhibited a larger downshift to 732 cm^–1^ in cells grown with ^13^C-bicarbonate relative to that observed with ^13^C-formate (Suppl Figure S9), suggesting that *R. eutropha*-GR preferentially uses carbon from ^13^C-bicarbonate over that from ^13^C-formate. We also quantified the newly synthesized proteins in single cells by calculating the area ratio of the 1655 to 1619 cm^–1^ bands and found higher amounts of these proteins with ^13^C-bicarbonate relative to ^13^C-formate (*p* < 0.0001) (Suppl Figure S9). These results suggest that *R. eutropha*-GR could also fix bicarbonate with formate acting as an organic electron donor. When formate acts as the carbon source, it is converted into CO_2_ and NADH by *R. eutropha* H16,^6^ with CO_2_ being fixed via the CBB cycle and NADH transformed into NADPH, via a proton-translocating transhydrogenase, to support cell growth.^20,37^ When extra energy is available via the light-harvesting GR, *R. eutropha*-GR fixed more CO_2_ from bicarbonate rather than from formate.

### Sources of Reductant for Light-Driven Autotrophic Growth of *R. eutropha*

A hybrid form of a photoautotrophic system was established to grow *R. eutropha*-GR (Suppl Figure S11) using CO_2_ and light as the inputs and biomass generation as the output. The light source serves two purposes: driving the solar panel to produce electricity for electron supply and activating intracellular GR in the cathode chamber to generate ATP (Suppl Figure S11).

**Figure 4:**
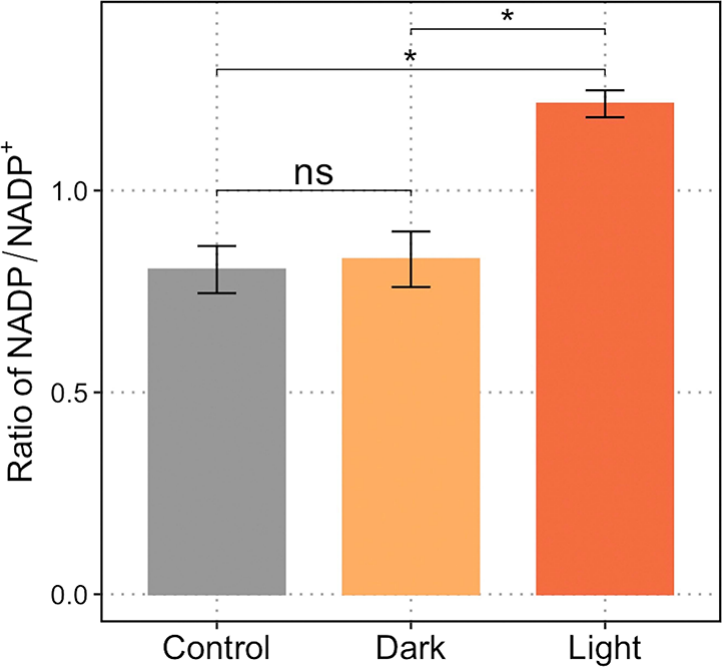
The intracellular NADPH/NADP^+^ ratios of *R. eutropha* H16 with pLO11a-blhDxrCRT-GR in different conditions. Independent *t* tests were performed: *p< 0.05. Data show means ± SD, *n* = 3. The NADPH/NADP^+^ ratio in the light was higher than that in the dark or a day 0 control.

**Figure 5:**
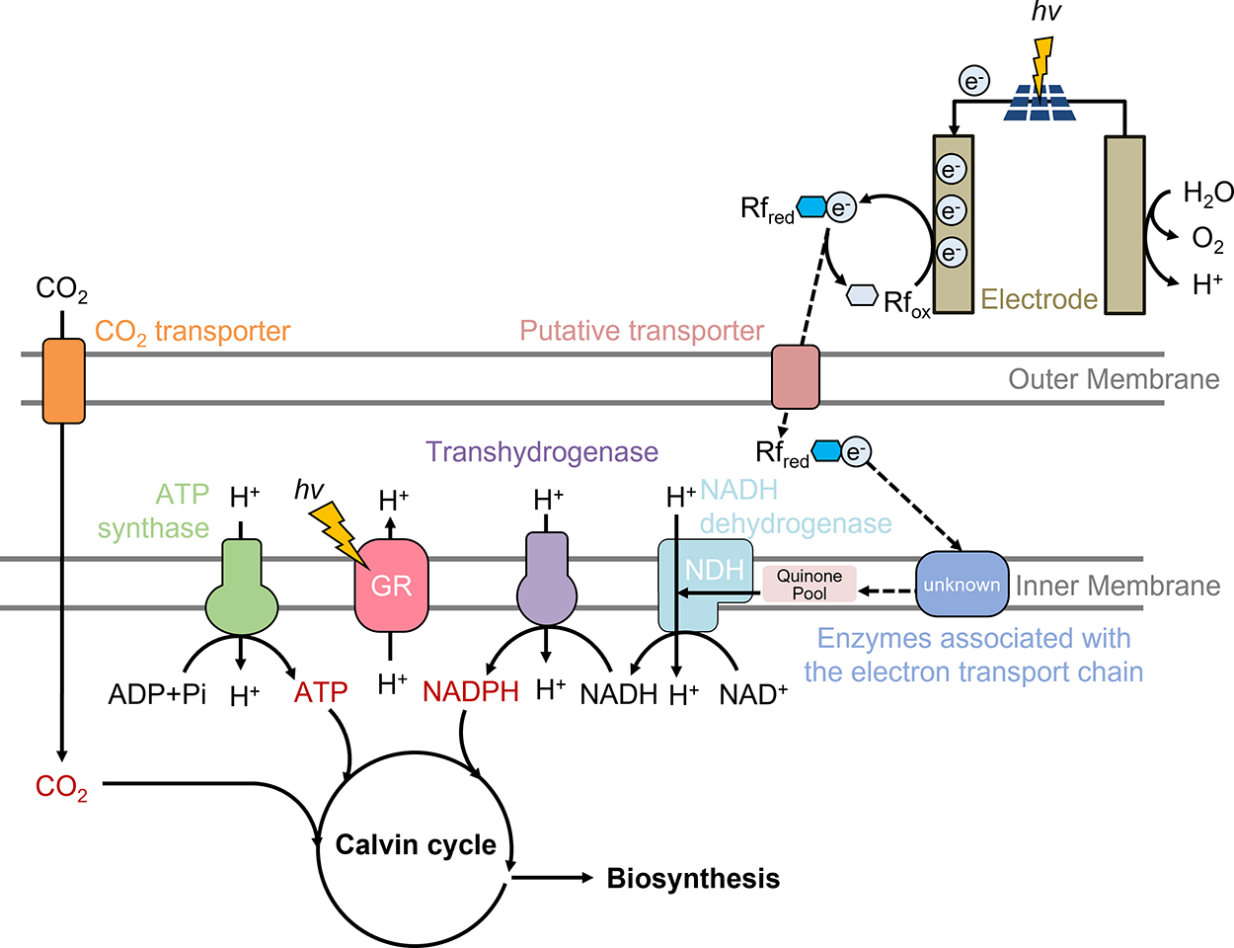
Photoelectrochemical CO_2_ fixation bioreactor system. A light source generates electricity, and electrons are transferred from electrode to cells mediated by riboflavin. The light-activated GR system with a complete redox loop can drive the autotrophic growth of bacteria via light-driven ATP synthesis. NADH is generated by an electron transfer from the quinol pool to NAD^+^ and is then converted into NADPH catalyzed by membrane-bound transhydrogenase. The mechanism by which riboflavin transfer electrons to *R. eutropha* is unclear and is represented by a dashed line.

Rather than using formate, a carbon cloth electrode can also be directly used as the electron donor for CO_2_ fixation, which requires an electron shuttle to transfer electrons from the cathode to the cells. We used riboflavin-mediated electron transfer from an electrode in a bioreactor with CO_2_ as the sole source of carbon. Riboflavin (−400 mV vs Ag/AgCl) is a common electron shuttling molecule used in microbial electrochemical systems.^38−40^ For some electroactive species, riboflavin interacts with cytochrome c and can transfer electrons from bacteria to an electrode;^40^ the reverse process, however, is thermodynamically unfavorable.^41^ Interestingly, we found that in this system riboflavin is able to transfer electrons from the cathode to bacterial cells when a light-driven proton pump (such as the GR) is present to overcome the energy hurdle. A microbial photoelectrochemical system was set up to confirm that GR-expressing cells are able to generate a reducing equivalent from electrode-supplied electrons in the presence of riboflavin and light. In the light, the NADPH/NADP^+^ ratio significantly increased compared to a control group and a dark group (Figure 4), indicating that NADPH can be synthesized in the absence of organic electron donors. Figure 5 illustrates the electron transport chain for the reductive fixation of CO_2_, which operates by reversing electron flow from the quinol pool to NADH dehydrogenase. In this electron transport chain, a proton-translocating transhydrogenase is required, which couples the reduction of NADP^+^ by NADH to the inward translocation of protons across the cytoplasmic membrane.^37,42^ Thus, the transmembrane proton gradient created by the light-driven turnovers of GR enables electron shuttling via riboflavin, leading to NADPH formation and ATP production by the ATP synthase (Figure 5).

**Figure 6:**
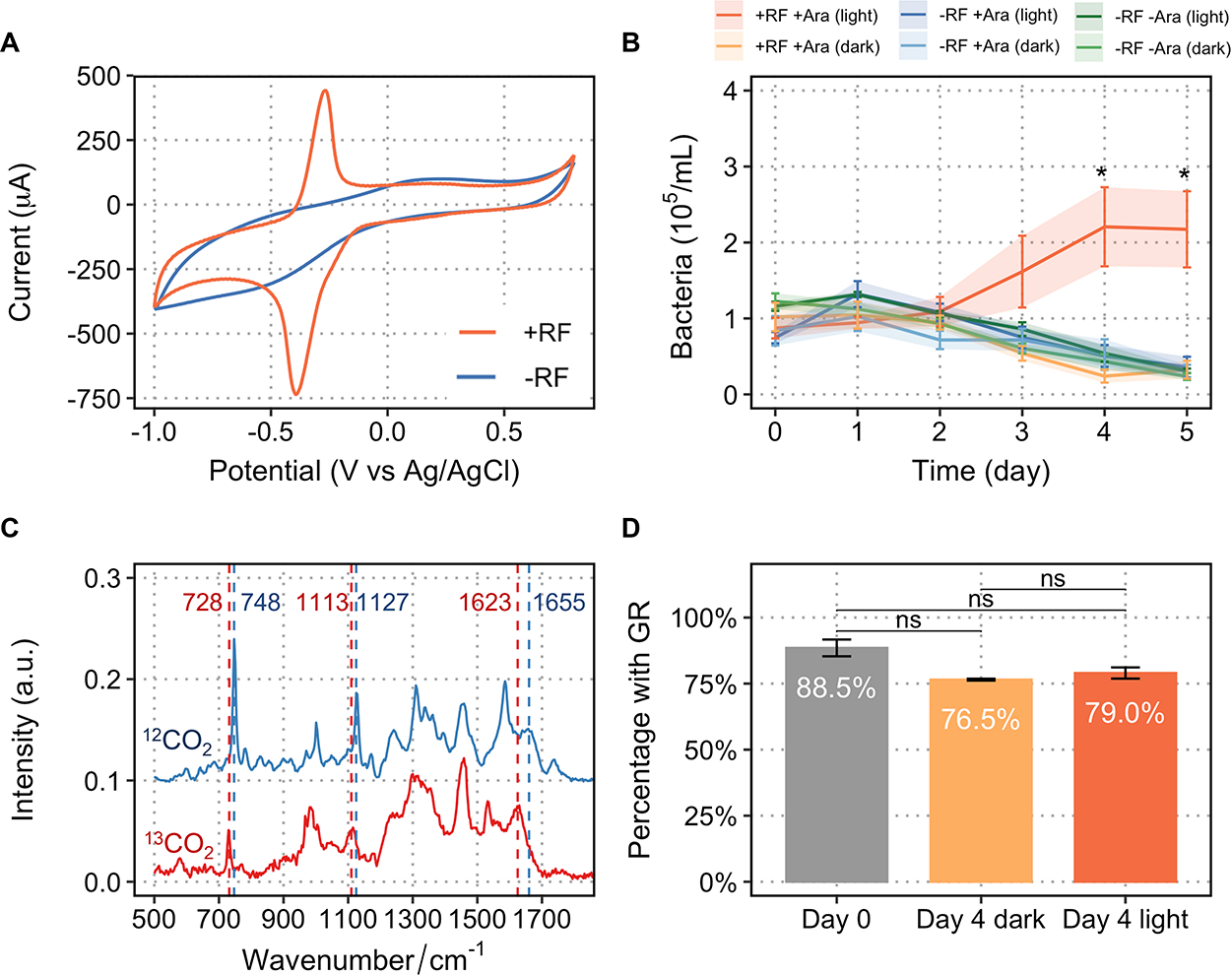
(A) Cyclic voltammetry (CV) analysis of the abiotic electrochemical system with or without 50 μM riboflavin using carbon cloth as the working electrode. CVs were conducted with a scan rate of 0.05 V/s within a potential range between −1.0 and 0.8 V versus Ag/AgCl electrode. (B) Growth of engineered *R. eutropha*-GR with CO_2_ as the sole carbon source, driven by light-activated GR and electricity (independent *t* tests were performed: *p < 0.05, **p < 0.01. Data show means ± SD, *n* = 3). (C) SCRS of cells of photoelectroautotrophic *R. eutropha*-GR grown on ^12^C-CO_2_ (blue) and ^13^C-CO_2_ (red). Cells grown on ^13^C-CO_2_ exhibited isotopic Raman shifts from 1655 to 1623 cm^–1^ (amide I of proteins), 1127 to 1113 cm^–1^ (cytochrome c), and 748 to 728 cm^–1^ (cytochrome c). (D) Bar chart showing the percentage of *R. eutropha*-GR cells that maintained high levels of GR after 4 days of incubation in the light and dark, without antibiotic selection pressure.

The reversible redox reactions of riboflavin were used to shuttle electrons from the electrode to the cell. We used cyclic voltammetry (CV) to verify these redox reactions at the electrode, which was made from carbon cloth. The cyclic voltammogram in Figure 6A shows typical oxidative and reductive peaks, indicating the suitability of the electrode material and yielding a midpoint potential for riboflavin of −0.39 V versus an Ag/AgCl standard electrode (Figure 6A). It also shows that when carbon cloth is used as the cathode electrode without riboflavin, it could not transfer electrons.

We used the bioreactor to demonstrate that *R. eutropha*-GR could perform photoautotrophic growth in the presence of the −0.6 V [versus Ag/AgCl] cathode, driven by light-activated GR and in the absence of formate (Figure 6B). Several negative controls show there was no detectable growth in the dark, even though the electrode potential, riboflavin, and GR were available, or in uninduced cells lacking GR but with riboflavin and the electrode potential (Figure 6B), or in the absence of riboflavin (Figure 6B). Although *R. eutropha* is able to use H_2_ as an energy source and will grow in its presence, the lack of cell growth in the negative controls should rule out the possibility of H_2_ generation from the cathode. Electrosynthesis of H_2_ requires a sufficient voltage potential, but the potential of the cathode (−0.6 V versus Ag/AgCl) in this study is insufficient for carbon cloth without coating catalysts to produce H_2_.

The autotrophic growth of *R. eutropha*-GR in the bioreactor was assessed using cell counts (Figure 6C) and verified by isotope labeling with ^13^CO_2_ as the sole carbon source for 4 days (Figure 6C). SCRS was used to analyze 200 single cells of *R. eutropha*-GR, which revealed multiple Raman shifts arising from the integration of ^13^CO_2_ into cellular biomass. Raman bands corresponding to cytochrome c at 748 (pyrrole breathing mode) and 1127 cm^–1^ (CN stretching vibrations) and a band arising from proteins at 1655 cm^–1^ (amide I) shifted to 728, 1113, and 1623 cm^–1^, respectively (Figure 6C). SCRS was also used to quantify the number of GR-containing bacterial cells from the bioreactor (Suppl Figure S9), which showed no significant change after 4 days (Figure 6D) and likely reflects a selective advantage conferred by GR, despite the absence of antibiotics.

The overall voltage to drive the photo-electrosynthetic growth of *R. eutropha*-GR in the bioreactor was 1.6–1.8 V. The corresponding light intensity on the solar panel was only 2 μmol m^–2^ s^–2^ (Suppl Figure S12), suggesting the bioreactor could operate in weak daylight. During the exponential growth phase (days 3–4 in Figure 6C), the maximal electron conversion efficiency from electricity to biomass was 20%.

Collectively, the results suggest that *R. eutropha*-GR can utilize light as an energy source to drive the CO_2_ fixation pathway for cell growth, in effect converting *R. eutropha* from chemolithoautotrophy to a new growth mode that is a hybrid form of photoautotrophy that could be termed photoelectroautotrophy.

## Discussion

The results of this study confirm that *R. eutropha*-GR can be engineered to grow autotrophically, using light to supply energy for GR to pump protons and for riboflavin to mediate electron transfers (Figure 5C). Thus, *R. eutropha* has been engineered to perform a type of hybrid photosynthesis, in which GR acts as a light-driven proton pump. The study supports the assumption that the proton gradient from GR was able to reverse the NADH dehydrogenase activity to make NADH from NAD^+^. PR-mediated NADH dehydrogenase reversion has previously been reported in *Shewanella oneidensis* MR-1,^43^ and in this study, we show that it can also occur in *R. eutropha.* The resulting proton motive force drives the transhydrogenase that forms NADPH, used for reductive fixation of CO_2_, as well as driving ATP synthase to produce ATP. Electrons required for the reductive assimilation of CO_2_ using the native CBB cycle can be provided by either an electrode (mediated by riboflavin) or an organic compound such as formate (Figure 1). Accordingly, biomass production in the light can be enhanced by 20% when formate is used as the electron donor (Figure 3A), but biomass can also be produced in a bioreactor with a CO_2_ carbon supply and a light source as the only inputs (Figure 6B). The biomass of *R. eutropha* H16 is a good source of single-cell protein for animal feed due to its high protein content, and it has been awarded a qualified presumption of safety (QPS) in the EU.^18,19^

The results show that riboflavin can mediate electron transfer between cells and the electrode. Although the mechanism of riboflavin delivering the electrons to the electron transport chain is still unknown, we have shown that riboflavin is able to serve as an electron-shuttling molecule to transfer electrons between *R. eutropha* and an electrode (Figure 6). We speculate that *R. eutropha* has a putative transporter for riboflavin-like molecules to enable riboflavin to pass through the outer membrane. Riboflavin is oxidized by enzymes that could be associated with the electron transport chain. A series of enzymes involved in the electron transport chain have been reported relevant to the electron shuttle-mediated electron transfer of *R. eutropha*. For example, the hydrogenase in *R. eutropha* is able to interact reversibly with electron shuttles such as flavin.^44^ The nitrate respiration chain was also upregulated in the presence of an electron mediator.^45^

The conversion of *R. eutropha* H16 from chemolithoautotrophy to a hybrid form of photoautotrophy has required the introduction of β-carotene biosynthesis, via the insertion of a four-gene biosynthetic pathway and overexpression of the *dxr* gene, and its subsequent conversion into retinal by adding a gene encoding β-carotene 15 15′-dioxygenase. The introduction of the gene encoding the GR creates a photosynthetic system built from a single rhodopsin protein, compared to one that relies on a series of chlorophyll protein complexes. As GR has a pKa = ∼4.8,^46^ compared to PR that has a pKa = ∼7.5,^47^ GR is functional at a lower pH, a situation often found in *R. eutropha* H16 growing using formate or CO_2_ as the sole carbon source. GR is originally from thylakoid-less *Gloeobacter violaceus* PCC7421;^48^ its high efficiency of proton pumping and rapid photocycle is probably to compensate for its reduced energy generation as compared to that from chlorophyll-based photosynthesis.^25^ It has been reported that GR expression in *E. coli* has increased biomass growth in the light^49^ and GR also has a higher molecular proton pumping rate than PR.^50^ However, the excess energy resulting from GR activity could also lead to the overproduction of ROS. It has been reported that GR-expressing *E. coli* showed elevated energy (ATP) levels but also increased ROS under aerobic conditions, resulting in a slower growth rate.^31^ Therefore, in this study, micro-aerobic conditions were used for the growth experiments. In the microbial photoelectrochemical system, oxygen limitation is an even more critical factor when taking into account both the ROS associated with the GR function and the cathode-derived ROS.^21^ As a result, the cathode chamber was only bubbled with CO_2_ to create anoxic conditions, which will limit both biomass growth and retinal production from β-carotene as the 15,15′-dioxygenase enzyme requires oxygen. In addition, riboflavin could be another limiting factor for biomass growth, possibly because of the low electron delivery rates directed from riboflavin to *R. eutropha* cells. Despite the low biomass growth rate, this study is the first proof of concept for rhodopsin-based photoelectrosynthesis.

In this study, we demonstrate that we can design a light-dependent electron transfer chain to drive photo-electrosynthesis using a rhodopsin-based system. Such a system only requires a rhodopsin and an electron mediator such as riboflavin. Microbial rhodopsin is a simple light-driven proton pump found broadly distributed in nature,^10,11^ and it can also be easily engineered into different bacterial hosts.^10,14,15^ Furthermore, GR has been shown to combine with other retinal analogues to absorb near-infrared light (850–950 nm),^51^ which can significantly extend the light-harvesting spectrum and maximize energy harvesting per surface area.^52^ The recyclable electron mediator riboflavin can be readily synthesized by bacteria^53^ or manually added into the reactor. Hence, the application of such a rhodopsin-based light-harvesting system would enable the conversion of various naturally heterotrophic bacteria into photoautotrophs that are able to use light for CO_2_ fixation. In natural oxygenic photosynthesis, a chlorophyll-based photosystem splits water and provides electrons to the redox reaction. The hybrid photoelectroautotrophic system shown here mimics photosynthesis using GR to generate a proton gradient and electricity as the electron donor. The result is that a new mode of photosynthetic growth has been engineered, enabling *R. eutropha* H16 to use solar energy to convert CO_2_ into biomass.

## Materials and Methods

### Bacterial Strains and Culture Conditions

*E. coli* strains were grown in LB broth at 37 °C under aeration by shaking at 200 rpm. *R. eutropha* strains were grown in Luria–Bertani (LB) broth at 30 °C under aeration by shaking at 150 rpm. If required, antibiotics (Sigma-Aldrich) were added as follows: 10 μg mL^–1^ gentamicin, 10 μg mL^–1^ tetracycline, 400 μg mL^–1^ kanamycin, and 500 μg mL^–1^ ampicillin for *R. eutropha*; 12.5 μg mL^–1^ tetracycline and 50 μg mL^–1^ kanamycin for *E. coli*. Induction of strains transformed with the pLO11a expression vector containing the arabinose-inducible P_BAD_ promoter was carried out by growth to log phase and the addition of 0.1% (w/v) l-arabinose (Sigma-Aldrich) and overnight growth at 30 °C for *R. eutropha* and 0.2% (w/v) l-arabinose and overnight growth at 37 °C for *E. coli*. Where required, induction of GR expression was accompanied by the addition of exogenous *trans*-retinal (Sigma-Aldrich) to a final concentration of 5 μg mL^–1^.

All constructs were assembled and expressed in a commercially obtained, chemically competent *E. coli* JM109 strain (Promega, UK). Two gentamicin-resistant *R. eutropha* strains, namely, H16 (ATCC 17699) and its derivative strain RHM5 (gift from Min-Kyu Oh, Korea University, South Korea; hereafter H16Δ*pha*) in which the *phaCAB* operon encoding the genes required for the conversion of acetyl-CoA to PHB has been deleted,^54^ were used for the expression of constructs. Recombinant plasmids were transformed into the *E. coli* S17-1 strain,^55^ made chemically competent using standard techniques.^56^ For conjugative plasmid transfer into *R. eutropha*, 1 mL overnight culture pellets of *E. coli* S17-1 and *R. eutropha* were mixed together in 100 μL LB media and spot dried onto a LB-agar plate, left overnight at 30 °C, and streaked to single colonies on an LB-agar plate containing gentamicin and appropriate selection antibiotics.

### Plasmid Construction

Common cloning procedures were performed according to standard protocols.^56^ Polymerase chain reaction (PCR) was carried out using Q5 DNA polymerase (NEB, UK) and synthesized primers (Sigma-Aldrich) according to the manufacturer’s instructions, and all PCR products were checked by DNA sequencing (Eurofins, Germany). A list of the plasmids and primers used in this study are shown in Table 1 and Supplementary Table S2, respectively, and details of plasmid construction are given in the Supporting Information.

### SCRS Measurements and Analysis

Bacterial cells were washed three times with distilled water to remove traces of culture medium prior to measurements. Samples were diluted until individual bacterial cells could be observed under a 100 × /0.75 microscope, and a 1.5 μL suspension was dropped onto an aluminum-coated slide and air dried. SCRS were obtained using a 532 nm neodymium–yttrium aluminum garnet laser with a 300 groove mm^–1^ diffraction grating (LabRAM HR Evolution, HORIBA, UK) and were acquired in the range of 100–3200 cm^–1^. The laser power was set at ∼30 mW, which was attenuated by neutral density (ND) filters before focusing onto the samples. For the measurements of the *Gloeobacter violaceus* PCC7421 rhodopsin (GR) complexes, 1% power filter and 1-second acquisition time were used; the low power and short acquisition time were used to prevent photobleaching of the chromophores. Each condition was measured with two biological replicates; each replicate was measured with more than 150 and 100 single cells in induced and uninduced samples, respectively, or 200 and 400 single cells before and after growth, respectively, under micro-aerobic conditions. Cells with GR complexes were identified by a band at ∼1530 cm^–1^. Intracellular PHB was identified and quantified by the band at 1735 cm^–1^.^33^ Raman bands at 748 (pyrrole breathing mode), 1128 (υ(CN) stretching vibrations), 1312 (δ (CH) deformations), and 1584 cm^–1^ (υ(CC) skeletal stretches) were used for quantifying the resonance Raman spectrum of cytochrome c, of which the vibrational modes with the porphyrin π–π* transitions are in resonance with the incident Raman laser at 532 nm, thereby greatly enhancing the signals.^33,57^ As in the measurements for determining the utilization of formate, bicarbonate, and carbon dioxide, 25% power filter and a 3- to 5-second acquisition time were used to acquire spectra with high signal-to-noise ratios. Each condition was measured with two to three biological replicates, each with more than 30 to 50 single cells acquired. Spectra were recorded with LabSpec 6 software (HORIBA, UK). All raw spectra were pre-processed by cosmic ray correction, polyline baseline fitting and subtraction, and vector normalization of the entire spectral region. Quantification of biomolecules was done by integrating the area of the corresponding Raman bands. All analysis and plotting were done under a R 4.0.0 environment.

### Growth of *R. eutropha* Strains under Light and Dark

Growth characterization experiments of *R. eutropha* with pLO11a-blhDxrCRT-GR were conducted under light and dark conditions, with and without induction, respectively. Before the experiments, *R. eutropha* strains harboring the plasmid were pre-cultivated overnight in tryptic soy broth (TSB) medium (17 g/L casein peptone, 2.5 g/L dipotassium hydrogen phosphate, 2.5 g/L glucose, 5 g/L sodium chloride, 3 g/L soya peptone) with 10 μg/mL of tetracycline, 5 μg/mL of *trans*-retinal, and 0.2% (w/v) l-arabinose at 30 °C under aeration by shaking at 150 rpm. After TSB preculture, cells were harvested by centrifugation at 3000 g for 5 min. The supernatant was discarded, and cells were washed three times with minimal medium (MM, 6.74 g/L Na_2_HPO_4_·7H_2_O, 1.5 g/L KH_2_PO_4_, 1.0 g/L (NH_4_)_2_SO_4_, 1 mg/L CaSO_4_·2H_2_O, 80 mg/L MgSO_4_·7H_2_O, 0.56 mg/L NiSO_4_·7H_2_O, 0.4 mg/L ferric citrate, 200 mg/L NaHCO_3_, and pH 7.0).^41^ Cells were grown under six different conditions: MM with and without 80 mM formate in micro-aerobic environments that were created in 15 mL tubes filled with 12 mL medium; MM containing 0.2% (w/v) l-arabinose as the inducer, with and without formate in micro-aerobic environments. Neither antibiotics nor exogenous *trans*-retinal was added in any of these conditions. Each growth condition was illuminated with a white LED light (∼50 μmol/s/m^2^) and dark wrapped in foil. In total, six growth conditions were analyzed with each condition having three replicates. Cells were initially resuspended in the MM to a final optical density (OD) of ∼0.01 and grown at 30 °C with shaking at 150 rpm.

### Bicarbonate Utilization in *R. eutropha* Growth

*R. eutropha* with pLO11a-blhDxrCRT-GR was grown in the MM containing 0.2% (w/v) l-arabinose micro-aerobically, using 40 mM formate and 40 mM bicarbonate as carbon sources. In order to investigate inorganic carbon fixation and the impact of bicarbonate utilization on biomass synthesis, ^13^C isotope-labeled formate and bicarbonate were used for growth experiments under the following isotopic conditions: (1) ^12^C-formate + ^12^C-bicarbonate, (2) ^13^C-formate + ^12^C-bicarbonate, and (3) ^12^C-formate + ^13^C-bicarbonate. Each experiment was carried out under light or dark conditions, and no antibiotics were added. Single-cell Raman spectroscopic measurements were carried out, and SCRS were used for the isotopic analysis.

### Microbial Photoelectrochemical System Apparatus

A dual-chamber bioreactor (70 mL for each chamber) separated by a Nafion membrane (only allowing proton transfer) was used as a bio-photoelectrochemical system for microbial growth experiments. For the anode chamber, carbon cloth (H23, 95 g m^–2^; 2.5 × 4.0 cm^2^; Quintech, Gloucestershire, UK) with a platinum catalyst (1 mg cm^–2^, PtC 60%; Fuel Cell Store) was used as the counter electrode. For the cathode chamber, the working electrode was made of 3.0 × 3.0 cm^2^ carbon cloth and an Ag/AgCl reference electrode (3 M KCl, RE-5B, BASi, USA) was installed for measuring the potentials. MM (as above) was added in both chambers. For the test of reducing power generation from electrode-supplied electrons, *R. eutropha* with pLO11a-blhDxrCRT-GR was grown overnight in MM with 80 mM formate, and the culture cells were collected and washed as above with the MM to give an initial OD_600_ of ∼0.1 before being injected into the cathode chamber. An electron shuttle (50 μM riboflavin) was then added to the chamber as the electron mediator. A polycrystalline solar panel (1.5 W, 140 mm × 180 mm, RS, UK) was used to generate electricity powered by light. Photovoltaic characterization of the solar panel is shown in Suppl Figure S11. The potential of the working electrode was controlled by a designed potentiostat. In the experiments, the potential was kept at −0.6 V [versus Ag/AgCl]. The cathode chamber was first bubbled with N_2_ to remove oxygen and then illuminated with white LED light (∼50 μmol/s/m^2^), operated at 30 °C, and agitated at 150 rpm. Cells were maintained overnight and collected for measurement of NADPH levels using a NADP^+^/NADPH quantification kit (Sigma-Aldrich). NADP_total_ and NADPH were extracted and quantified according to the manufacturer’s protocol. The amount of NADP^+^ was calculated by the difference between NADP_total_ and NADPH, and then the NADPH/NADP^+^ ratio was obtained. Figure S10 shows the calibration curve between NADPH amount and absorbance at 450 nm.

For the test of light-driven autotrophic growth, *R. eutropha* with pLO11a-blhDxrCRT-GR was grown overnight in TSB and pretreated as above with the MM to give an initial OD_600_ of ∼0.01 before being injected into the cathode chamber. The cathode chamber was bubbled with CO_2_ at a flow rate of 20 mL/min, illuminated with white LED light (∼50 μmol/s/m^2^), operated at 30 °C, and agitated at 150 rpm. Pictures of the microbial photoelectrochemical system apparatus can be found in Suppl Figure S11.

The electron transfer efficiency of the biomass formation could be calculated by the equation,
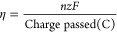
where *n* is the amount of biomass (using the classic formula C_5_H_7_NO_2_) product (mol), *z* is the number of transferred electrons (*z* = 4 for CO_2_ conversion to biomass), and *F* is the Faraday constant (96,485 C/mol). The average current is charge divided by time. In the solar panel system with riboflavin, the average electricity current was −11.2 μA under stable operation in the presence of GR-expressing cells and −9.7 μA in the presence of cells without induction.

### Counting Colony-Forming Units (CFUs) to Measure the Concentration of Viable Cells in the Cathode Chamber

The viable cells in the cathode chamber consist of the cells on the electrode and in the supernatant. For the counting of the cells attached on the electrode, the carbon cloth electrode was placed in a 50 mL test tube containing 5 mL of 0.9% sterile NaCl solution and vortexed for 2 min. A series of diluted solutions were spread onto LB agar plates, which were then incubated at 30 °C for 48 h, and the CFU was counted to estimate the total number of viable cells on the electrode. For the counting of the cells in the supernatant, 1 mL of mixture was collected from the cathode chamber and a series of diluted solutions were spread onto LB agar plates, which were then incubated at 30 °C for 48 h prior to CFU counting. In this way, the total number of viable cells in the cathode chamber (cells on the electrode plus cells in the supernatant) was calculated and then divided by the working volume to estimate the concentration of viable cells.

## Supporting Information Available

Materials, methods, and results on plasmid construction (PDF)

## Notes

All data discussed in the paper will be made available to readers.

## Notes

The authors have filed a provisional patent application with the UK Patent Office related to this work.
