# Supplementary material for: Engineering a Rhodopsin-Based Photo-Electrosynthetic System in Bacteria for CO2 Fixation
Source: ACS Synth Biol. 2022 Oct 20;11(11):3805–16. doi: 10.1021/acssynbio.2c00397 (PMC9680020; doi:10.1021/acssynbio.2c00397)
Supplement: Supplementary file 1 — sb2c00397_si_001.pdf [file sb2c00397_si_001.pdf]

Supplementary information

Engineering a Rhodopsin-based Photo-electrosynthetic System in Bacteria for CO_2 Fixation

Paul A. Davison [a#], Weiming Tu [b#] Jiabao Xu [b], Simona Della Valle [b], Ian P. Thompson [b], C. Neil Hunter [a] and Wei E. Huang [b*]

[a] Plants, Photosynthesis and Soil, School of Biosciences, University of Sheffield, Sheffield, United Kingdom.

[b] Department of Engineering Science, University of Oxford, Oxford, United Kingdom.

[#] W.T. and P.A.D contributed equally to this work.

[*]Corresponding author: Department of Engineering Science, University of Oxford, Parks Road, OX1 3PJ, Oxford, United Kingdom. Email wei.huang@eng.ox.ac.uk Tel: +44 1865 283786.

Paul A. Davison − Plants, Photosynthesis and Soil, School of Biosciences, University of Sheffield, Sheffield S10 2TN, United Kingdom.

Weiming Tu − Department of Engineering Science, University of Oxford, Oxford OX1 3PJ, U.K.

Jiabao Xu − Department of Engineering Science, University of Oxford, Oxford OX1 3PJ, U.K.

Simona Della Valle − Department of Engineering Science, University of Oxford, Oxford OX1 3PJ, U.K.

Ian P. Thompson − Department of Engineering Science, University of Oxford, Oxford OX1 3PJ, U.K.

C. Neil Hunter − Plants, Photosynthesis and Soil, School of Biosciences, University of Sheffield, Sheffield S10 2TN, United Kingdom.

Wei E. Huang − Department of Engineering Science, University of Oxford, Oxford OX1 3PJ, U.K.

Running title: Engineering photoautotrophy in *Ralstonia eutropha* H16

Materials and methods

Plasmid construction

The detailed information about primers used in this study is shown in Supplementary Table S1.

A modified version of the pLO11 (Tc^r, RK2 *ori*, Mob^+) vector specifically designed for expression in *R. eutropha* (*1*), with the acetoin promoter replaced by the *araC* gene and the arabinose inducible P_{BAD} promoter (henceforth called 'pLO11a') was used for the expression of heterologous genes (gift from Oliver Lenz, Technische Universität Berlin, Germany).

The *crtY* and *crtI* genes were isolated by PCR as a single fragment from the pRER1B plasmid that contains the *crtY* and *crtI* genes from *Erwinia herbicola* (*Pantoea agglomerans)* (*2*) using the primers CrtYI_F and CrtYI_R and cloned into the *Nco*I-*Hin*dIII sites of pLO11a to create pLO11a-CrtYI.

The *Erwinia uredovora* (*Pantoea ananatis) crtEXYIB* operon responsible for conversion of GGPP to β-carotene in *E. coli,* including the 879 bp upstream region required for constitutive β-carotene expression, was isolated by PCR as a single fragment from the pORANGE (*3*) plasmid (gift from Kwang-Hwan Jung, Sogang University, S.Korea) using the primers CRT_F and CRT_R and cloned into the *Sac*I-*Bgl*II sites of pLO11a to create pLO11a-CRT.

The *dxr* gene from *R. eutropha* (GenBank: CP039287.1) was synthesised (Integrated DNA Technologies, USA) and isolated by PCR using the primers SacDxr_F and SacDxr_R and cloned into the *Sac*I site of the pLO11a-CRT plasmid to create plasmid pLO11a-DxrCRT. It was also isolated by PCR using the primers NcoDxr_F and HindDxr_R and cloned into the *Nco*I-*Hind*III sites of pLO11a to create pLO11a-Dxr.

The gene encoding the *Gloeobacter violaceus* PCC7421 rhodopsin, GR, (gift from Kwang Hwan-Lung, Sogang University, S.Korea; Genbank: BA000045) was isolated by PCR using the primer pair GR_F and GR_R and cloned into the *Nco*I and *Bgl*II sites of pLO11a.

The β-carotene 15, 15'-dioxygenase gene (*blh*) from the uncultured marine bacterium 66A03 (GenBank: DQ065755.1) previously shown to produce retinal in *E. coli* (*4*) was codon optimised for *R. eutropha* and synthesised (Integrated DNA Technologies, USA) flanked by *Nco*I-*Hind*III sites and cloned into pLO11a to create pLO11a-blh. The *blh* gene with an upstream ribosome binding site was created by PCR from pLO11a-blh using primers Rbsblh_F and LOterm_R and inserted into the *Hind*III site of pLO11a-Dxr to create pLO11a-Dxr-blh. The *dxr-blh* operon was created by PCR from pLO11a-Dxr-blh using primers Dxr_F and

Blh_R and inserted into the *Hind*III site of pLO11a-CRT to create pLO11a-blhDxrCRT. The *GR* gene with its own P_{BAD} promoter was created by PCR from pLO11a-GR using primers pBAD_F and LOterm_R and inserted into the *Eco*RI site of pLO11a-blhDxrCRT to create pLO11a-blhDxrCRT-GR.

Carotenoid extraction and analysis

Strains transformed with the pLO11aa expression vector constructs were grown in 50ml LB with 12.5 µg ml^{-1} tetracycline to log phase and either induced with 0.1% (w/v) L-arabinose (Sigma-Aldrich) and overnight growth at 30 °C for *R. eutropha* or 0.2% (w/v) L-arabinose and overnight growth at 37 °C for *E. coli*. Carotenoids were solvent extracted from the 50ml cell culture pellets with 1 ml of 7:2 acetone:methanol (v/v), clarified by centrifugation and an absorption spectrum taken using an Agilent Cary 60 UV-Vis spectrophotometer. For more detailed analysis the solvent extract was dried down under nitrogen, re-dissolved in methanol and carotenoids separated by reversed-phase HPLC on an Agilent 1100 HPLC system using a Supelco Discovery HS C18 column as previously described (*5*). Elution of β-carotene and retinal were monitored at 450 nm and 380 nm respectively. An all *trans*-β-carotene standard (Sigma-Aldrich) of known concentration was used for the quantification of integrated peak areas. A pure all *trans*-retinal standard (Sigma-Aldrich) was used to assign the retinal peak.

Membrane purification

A 20 ml LB overnight culture with appropriate antibiotic selection of *R. eutropha* was used to inoculate 500 ml LB with selection in a 2.5 L conical flask. This was grown at 150 rpm and 30ºC to OD_{600} = 0.5 - 07, induced with 0.1% (w/v) L-arabinose and 5 µg ml^{-1} all *trans*- retinal and grown overnight at 30ºC. The culture was pelleted and resuspended in approximately 10 ml of buffer A (25 ml K_2HPO_4/KH_2PO_4 pH 7.4) and lysed by two cycles of French pressing at a pressure of 18 000 psi. Membrane fractions were isolated on a 10-50% continuous sucrose gradient (the sucrose was made up in buffer A and 1.5 ml broken cells loaded per gradient) spun at 30,000 rpm for 2 hours at 4°C. A 1 ml membrane fraction was removed and an absorption spectrum taken using an Agilent Cary 60 UV-Vis spectrophotometer.

PHB accumulation from formate in the light and dark

The engineered *R. eutropha* precultured in TSB with 100 μg/mL kanamycin was centrifuged and washed three times with a nitrogen-limited minimal medium (3.57 g/L Na_2HPO_4, 1.5 g/L KH_2PO_4, 0.1 g/L $(NH_4)_2SO_4$, 0.08 g/L $MgSO_4{\cdot}7H_2O$, 0.2 g/L $NaHCO_3$, 1 mg/L $CaSO_4{\cdot}2H_2O$, 0.56 mg/L $NiSO_4{\cdot}7H_2O$, 0.4 mg/L ferric citrate, and pH 7.0) to remove TSB medium.

Subsequently, the cell pellet was resuspended in the nitrogen-limited minimal medium, and the OD was adjusted to 1 before being transferred into flasks with a total volume of 250 mL. Formate (80 mM) was added into baffled flasks with a working volume of 200 mL for micro-aerobic PHB synthesis. The flasks were illuminated with a white LED light (~50 µmol/s/m^2) and dark wrapped in foil. Unless otherwise stated, all cultures were grown at 30 °C and 150 rpm.

Results

Identification of genes for beta-carotene synthesis in R. eutropha H16

Analysis of the KEGG pathway for *R. eutropha* H16 (*Cupriavidus nectar* H16; https://www.genome.jp/kegg-bin/show_pathway?reh00906) shows that it contains homologues of *crtE* and *crtB* (Genbank accession numbers CAJ92601.1 and CAJ93812.1 respectively) but not *crtI* and *crtY*. To see whether these homologous *crtE* and *crtB* genes were functional, and their products could be combined with exogenous CrtY and CrtI enzymes to make β-carotene, the *crtY* and *crtI* genes were isolated as one fragment by PCR from the *crt* operon of *Erwinia herbicola*, where they are situated adjacent to each other in the *crt* operon, and inserted into the modified pLO11 (Tc^r, RK2 *ori*, Mob^+) vector containing the arabinose inducible P_{BAD} promoter (pLO11a) specifically designed for expression in *R. eutropha* (*1*). This plasmid pLO11a-CrtYI (Table 1) was transformed into wild-type *R. eutropha* H16 by conjugation, the subsequent strain grown to log phase in LB with 12.5 µg ml^{-1} tetracycline, grown overnight in the presence and absence of arabinose and the cell pellets subjected to solvent extraction and HPLC analysis. No β-carotene could be detected by HPLC in either the negative control consisting of *R. eutropha* H16 transformed with the empty pLO11a vector or the strain containing pLO11a-CrtYI in either the absence or presence of arabinose (results not shown). This suggests that the native *crtE* and *crtB* genes in *R. eutropha* H16 are not functional in the β-carotene biosynthetic pathway.

The pORANGE plasmid, which gives constitutive β-carotene expression in *E. coli* JM109 (*3*), was used in attempts to synthesise this carotenoid in *R. eutropha* H16. DNA sequencing revealed that this plasmid encodes the carotenoid biosynthesis gene cluster (*crtE*, *crtX*, *crtY*, *crtI*, *crtB;* the *crtX* gene encodes the zeaxanthin glucosyltransferase enzyme which is not involved in β-carotene synthesis) from *Pantoea ananatis* (*Erwinia uredovora*) and an 879 bp region upstream of *crtE* that contains the putative 240 bp promoter region (Genbank: D90087)

required for constitutive expression of this operon in *E. coli* JM109. The entire *crtEXYIB* operon, including the *crtE* promoter region (*crt* operon), was isolated by PCR from pORANGE and cloned into the pLO11a expression plasmid to create pLO11a-CRT (designated pCRT; plasmid map shown in Suppl Fig. S1A).

Overexpression of the dxr* gene *in R. eutropha enhanced β-carotene production

Isopentenyl diphosphate (IPP) is the common, five-carbon building block in the biosynthesis of all carotenoids with the second reaction in its synthesis being the reduction of 1-deoxy-D-xylulose 5-phosphate (DXP) to 2-C-methyl-D-erythritol-4-phosphate, catalysed by DXP reductoisomerase and encoded by *dxr* (Fig. 2A). Overexpression of the *Synechocystis dxr* gene in tobacco and the maize *dxr* gene in *Zea mays* has been shown to increase carotenoid content in tobacco and maize plants (*6, 7*). In order to try and increase the levels of β-carotene in *R. eutropha* H16 an attempt was made to overexpress the native *dxr* gene that encodes DXP reductoisomerase (EC:1.1.1.267), the homologue of *E. coli dxr*, in conjunction with the *crt* operon. The *dxr* gene was synthesised and cloned into the pLO11a-CRT plasmid to create plasmid pLO11a-Dxr-CRT (designated pDxrCRT; plasmid map shown in Suppl Fig. S1B) in which expression was induced by arabinose. When this construct was transformed into *E. coli* JM109 the cells turned yellow without arabinose induction (Suppl Fig. S2A), whilst transformed *R. eutropha* H16 cells only appeared slightly yellow in the presence of arabinose (Suppl Fig. S2B). Subsequent solvent extraction of *R. eutropha* H16 pDxrCRT cells and HPLC analysis showed that β-carotene was produced after induction (Suppl Fig. S2C, solid line), whilst no β-carotene was detectable in the absence of arabinose (Suppl Fig. S2C, broken line). A negative control strain of *R. eutropha* H16, containing only the *dxr* gene cloned by PCR into the pLO11a expression plasmid produced no β-carotene in the absence or presence of arabinose (results not shown).

Redirection of carbon flux promoted β-carotene production in R. eutropha

The pDxrCRT construct was transformed into the RHM5 Δ*phaCAB* strain (henceforth referred to as H16Δ*pha*) as it has been shown that deleting the *phaCAB* operon encoding the three metabolic pathway genes from acetyl-CoA to polyhydroxybutyrate (PHB) increases production in *R. eutropha* of ethanol from acetate (8). The resulting strain looked demonstrably more yellow after induction with arabinose (Suppl Fig. 2D) and β-carotene could easily be extracted into solvent and detected using an absorbance scan (Suppl Fig 2E) where the effect of induction on β-carotene production was clearly observable; subtraction of the uninduced

scan from the induced gave a difference spectrum identical to that of β-carotene (Suppl Fig. 4F); HPLC analysis confirmed the presence in the solvent extract of *trans*- and *cis*-β-carotene observed as peaks eluting at about 16.9 minutes and 17.1 minutes respectively (Suppl Fig. 2G).

Three weighed cell pellets from 50-ml cultures of *R. eutropha* H16 pCRT and H16 pDxrCRT were each solvent extracted, the extract analysed by HPLC to determine the mean integrated β-carotene peak areas (as determined by Agilent ChemStation HPLC software) and compared against the peak areas obtained from several dilutions of a 1 mg/ml β-carotene standard dissolved in methanol. Yield values for *R. eutropha* H16 pCRT and pDxrCRT of approximately 0.6 ± 0.2 μg and 0.9 ± 0.1 μg β-carotene per g wet weight of pellet respectively were obtained showing that more β-carotene was produced in the presence of *dxr*. A further improvement in yield was obtained with *R. eutropha* H16Δ*pha* pDxrCRT where a value of approximately 2 ± 0.5 μg β-carotene per g wet weight of pellet was obtained (Suppl Fig. 2H).

Validation of GR biosynthesis in E. coli and R. eutropha H16 using single cell Raman micro-spectroscopy

In order to determine the presence *in vivo* of the GR-retinal complex in cells expressing the *GR* gene, samples of uninduced and arabinose induced H16Δ*pha* GR, JM109 blhDxrCRT-GR and H16Δ*pha* blhDxrCRT-GR strains were examined at a single-cell level by Raman spectroscopy. Induction of H16Δ*pha* GR included the addition of exogenous retinal whereas induction of JM109 blhDxrCRT-GR, H16Δ*pha* blhDxrCRT-GR and H16 blhDxrCRT-GR did not as these strains make their own endogenous retinal. Single-cell Raman spectra (SCRS) were obtained ($n > 150$ in each induced sample and $n > 100$ in each uninduced sample) and the averaged spectra are shown in Suppl Fig. S4A. A prominent band at 1530 cm^{-1} was present in all induced samples of H16Δ*pha* GR (labelled H16-GR), JM109 blhDxrCRT-GR (labelled JM109) and H16Δ*pha* blhDxrCRT-GR (labelled H16); this band was also present in the H16 blhDxrCRT-GR strain (where the *pha* operon has not been deleted), appearing upon arabinose induction (Suppl Fig S4B). This characteristic Raman band was attributed to ethylenic stretching ($\nu_{C=C}$) vibrations in retinal-protein complexes (*9*), which depending on the carbon chain length and structure, can shift between 1505–1530 cm^{-1} (*10*). To date, Raman spectroscopy has only been carried out on purified GR protein either as crystals (*11*) or reconstituted into liposomes (*12*) where the ethylenic stretching ($\nu_{C=C}$) band was typically seen between 1524-1535 cm^{-1} depending on the pH conditions.

The percentage of cells expressing GR-retinal in a population was calculated by counting the number of SCRS that contained the 1530 cm^{-1} GR band (Table S2). The induced JM109 blhDxrCRT-GR sample had 41% of its population present with GR-retinal whilst 9% of the uninduced population also had GR-retinal present which is presumably a reflection of some 'leakiness' in the P_{BAD} promoter. Induced samples of H16Δ*pha* blhDxrCRT-GR and H16Δ*pha* GR showed 8% and 89% respectively of the populations expressing GR-retinal; no Raman band at 1530 cm^{-1} was observed in the uninduced samples. The percentage of cells expressing GR-retinal (Table S2) was highest in the H16Δ*pha* GR strain, to which exogenous retinal had been added, and is presumably a reflection of the reduced amount of endogenous retinal produced in *E. coli* JM109 and *R. eutropha* H16Δ*pha* by the pLO11a blhDxrCRT-GR construct, although the higher percentage of cells in the former indicates better expression of the construct in *E. coli*. As the amount of endogenous retinal is the limiting factor in the production of GR-retinal there is certainly more scope for improving its production, for example, through codon optimisation of all the genes, the use of different promoters with differing induction conditions and molecular evolution/screening techniques.

Supporting information references

1. A. Schwarze, M. J. Kopczak, M. Rogner, O. Lenz, Requirements for construction of a functional hybrid complex of photosystem I and [NiFe]-hydrogenase. *Appl Environ Microbiol* **76**, 2641-2651 (2010).
2. C. N. Hunter *et al.*, Introduction of New Carotenoids into the Bacterial Photosynthetic Apparatus by Combining the Carotenoid Biosynthetic Pathways of Erwinia-Herbicola and Rhodobacter-Sphaeroides. *J Bacteriol* **176**, 3692-3697 (1994).
3. S. Y. Kim, S. A. Waschuk, L. S. Brown, K. H. Jung, Screening and characterization of proteorhodopsin color-tuning mutations in Escherichia coli with endogenous retinal synthesis. *Biochim Biophys Acta* **1777**, 504-513 (2008).
4. Y. S. Kim, C. S. Park, D. K. Oh, Retinal production from beta-carotene by beta-carotene 15,15'-dioxygenase from an unculturable marine bacterium. *Biotechnol Lett* **32**, 957-961 (2010).
5. L. A. Malone *et al.*, Cryo-EM structure of the spinach cytochrome b(6) f complex at 3.6 angstrom resolution. *Nature* **575**, 535-+ (2019).
6. T. Hasunuma *et al.*, Overexpression of 1-Deoxy-D-xylulose-5-phosphate reductoisomerase gene in chloroplast contributes to increment of isoprenoid production. *J Biosci Bioeng* **105**, 518-526 (2008).
7. J. Hans, B. Hause, D. Strack, M. H. Walter, Cloning, characterization, and immunolocalization of a mycorrhiza-inducible 1-deoxy-d-xylulose 5-phosphate reductoisomerase in arbuscule-containing cells of maize. *Plant Physiol* **134**, 614-624 (2004).
8. Lee, H.M., Jeon, B.Y. & Oh, M.K. Microbial production of ethanol from acetate by engineered Ralstonia eutropha. *Biotechnol Bioproc E* **21**, 402-407 (2016).
9. Y. Z. Song *et al.*, Proteorhodopsin Overproduction Enhances the Long-Term Viability of Escherichia coli. *Appl Environ Microb* **86**, (2020).

10. J. Jehlicka *et al.*, Potential and limits of Raman spectroscopy for carotenoid detection in microorganisms: implications for astrobiology. *Philos T R Soc A* **372**, (2014).
11. T. Morizumi *et al.*, X-ray Crystallographic Structure and Oligomerization of Gloeobacter Rhodopsin. *Sci Rep-Uk* **9**, (2019).
12. M. R. M. Miranda *et al.*, The Photocycle and Proton Translocation Pathway in a Cyanobacterial Ion-Pumping Rhodopsin. *Biophys J* **96**, 1471-1481 (2009).

Supplementary Tables and Figures

Table S1. The percentage of cells with GR in *Ralstonia eutropha* H16 blhDxrCRT-GR under microaerobic or aerobic conditions before and after growth for 72 h without antibiotics.

Conditions	Total cells measured by Raman	Cells with GR	Percentage
0 h	200	165	82.5%
Microaerobic 72 h light	400	277	69.2%
Microaerobic 72 h dark	400	148	36.8%
Aerobic 72 h light	400	86	26.2%
Aerobic 72 h dark	400	143	35.8%

Table S2 – primers used in this study

Primer name	Sequence
CrtYI_F	GATCCATGGGAAGGGATCTGATTTTAGTCGGC
CrtYI_R	GATAAG CTTGCTCATTGCAGATCCTCAATCA
CRT_F	GCTCTAGAGCTCGATCTCAAACAGGATTGGGC
CRT_R	GCTCTAGATCTGACGCTCCGGGAAAGAC
SacDxr_F	GCGGAGCTCATG CATCGCATTACCATCCTG
SacDxr_R	GCGGAGCTCTCAGCGCTTTGCAGCC
NcoDxr_F	GCACCATGGGCCATCGCATTACCATCCTGGG
HindDxr_R	GCAAAGCTTCAGCGCTTTGCAGCC
pBAD_F	GCAGATCTGAATTCAAGAAACCAATTGTCCATATTGCATC
LOterm_R	GCTGAATTCAGATCTGTGACGCAGTAGCGGTAAACG
GR_F	CATGCCATGGGTTTGATGACCGTATTTTCTTC
GR_R	GCAGATCTGCGGCCGCTAGGAGATAAGACTGCCTCCCG
Rbsblh_F	GCGAAGCTTAAGGAGGAGACCCCATGG
Dxr_F	GCGGAGCTCATGGGCCATCGCATTACCATC
Blh_R	GCGGAGCTCTTATTAATGGTGATGGTGATGATGGC

Supp. Fig S1

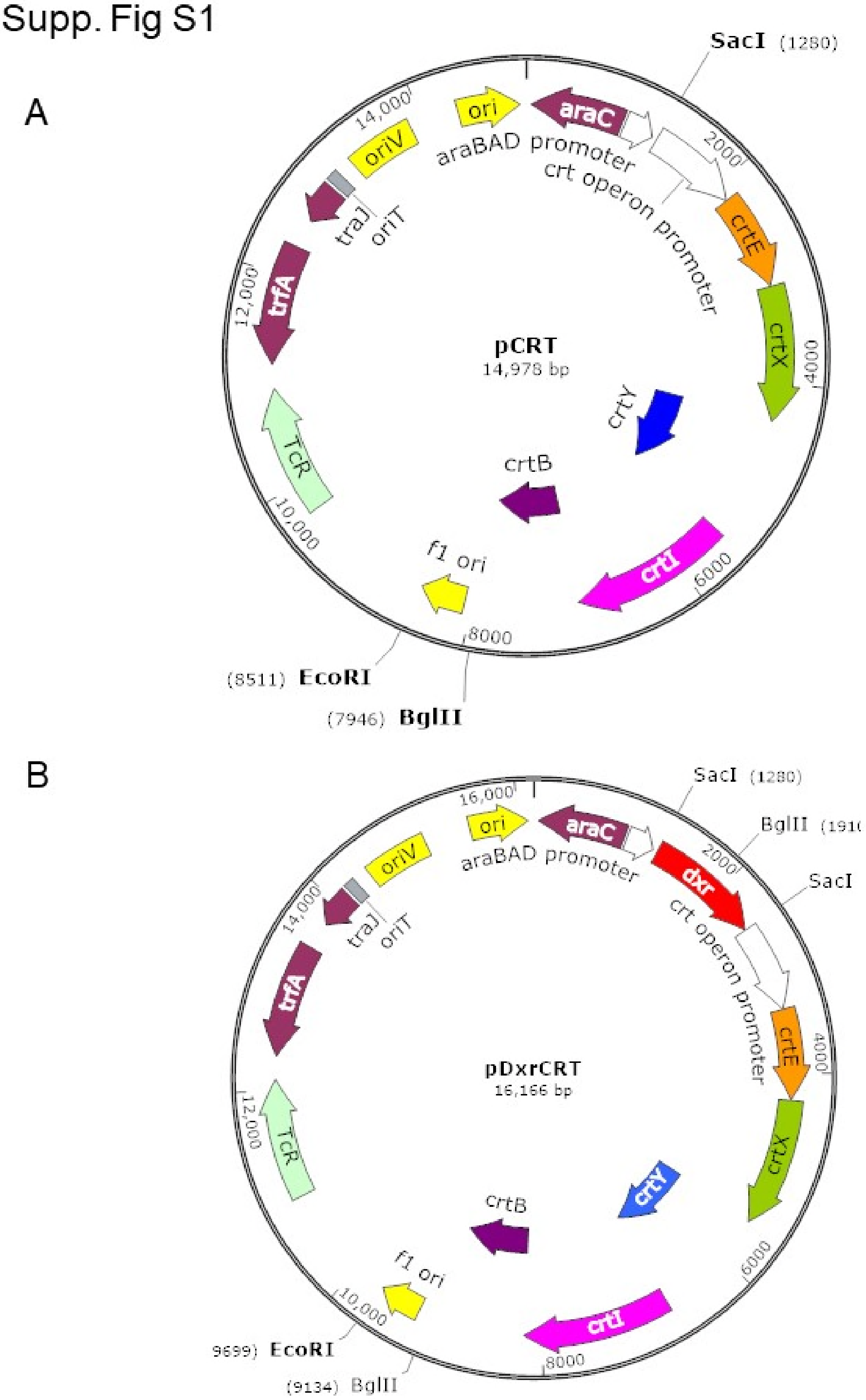

Supplementary figure S1 Plasmid maps of (A) pCRT and (B) pDxrCRT. The genes with their associated proteins are as follows: dxr, 1-deoxy-D-xylulose 5-phosphate reductoisomerase; crtE, geranylgeranyl diphosphate synthase; crtX, zeaxanthin glucosyltransferase; crtB, phytoene synthase; crtI, phytoene desaturase; crtY, lycopene cyclase. The plasmid backbone in both cases is pLO11a.

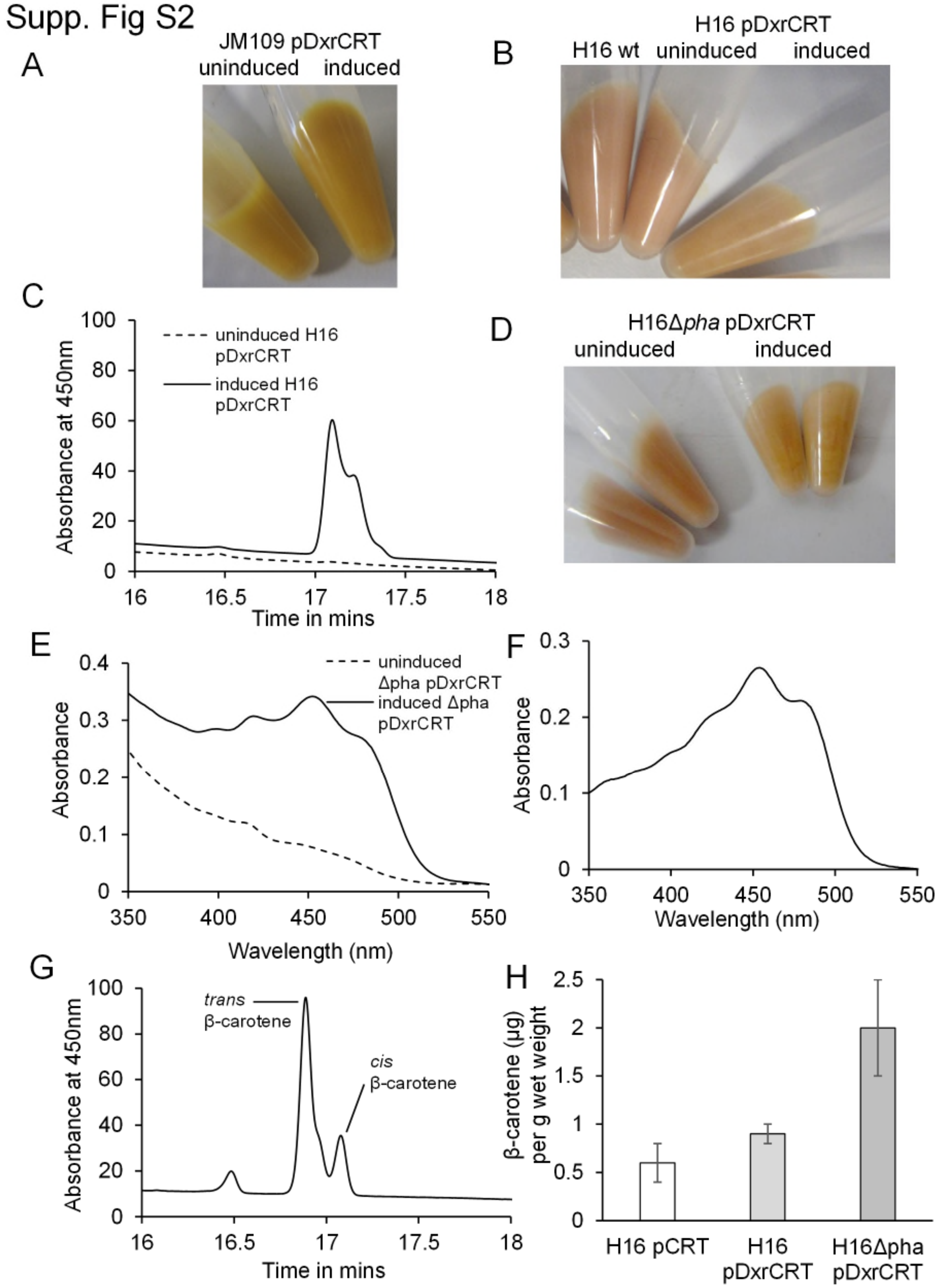

Supplementary figure S2 (A) Cell pellets of 50 ml cultures of *E. coli* JM109 transformed with the pLO11a-Dxr-crt (pDxrCRT) construct grown in the absence (uninduced) and presence (induced) of arabinose. (B) Cell pellets of 50 ml cultures of *R. eutropha* H16 wild-type cells

(wt) and cells transformed with pDxrCRT grown in the presence and absence of arabinose. (C) HPLC elution profiles at 450nm of solvent extracted uninduced (broken line) and induced (solid line) *R. eutropha* H16 pDxrCRT cells. (D) Cell pellets of 50 ml cultures of *R. eutropha* H16Δ*pha* cells transformed with the pDxrCRT construct grown in the absence (uninduced) and presence (induced) of arabinose. (E) Absorbance scan of solvent extracted uninduced (broken line) and induced (solid line) H16Δ*pha* pDxrCRT cells and the resulting difference absorbance scan (F). (G) HPLC elution profile at 450nm of solvent extracted induced H16Δ*pha* pDxrCRT cells with the *tran*s- and *cis*-β-carotene peaks indicated. (H) Yield of β-carotene (expressed as µg per g of wet weight pellet) in induced *R. eutropha* H16 pCRT, H16 pDxrCRT and H16Δ*pha* pDxrCRT.

Supp. Fig S3

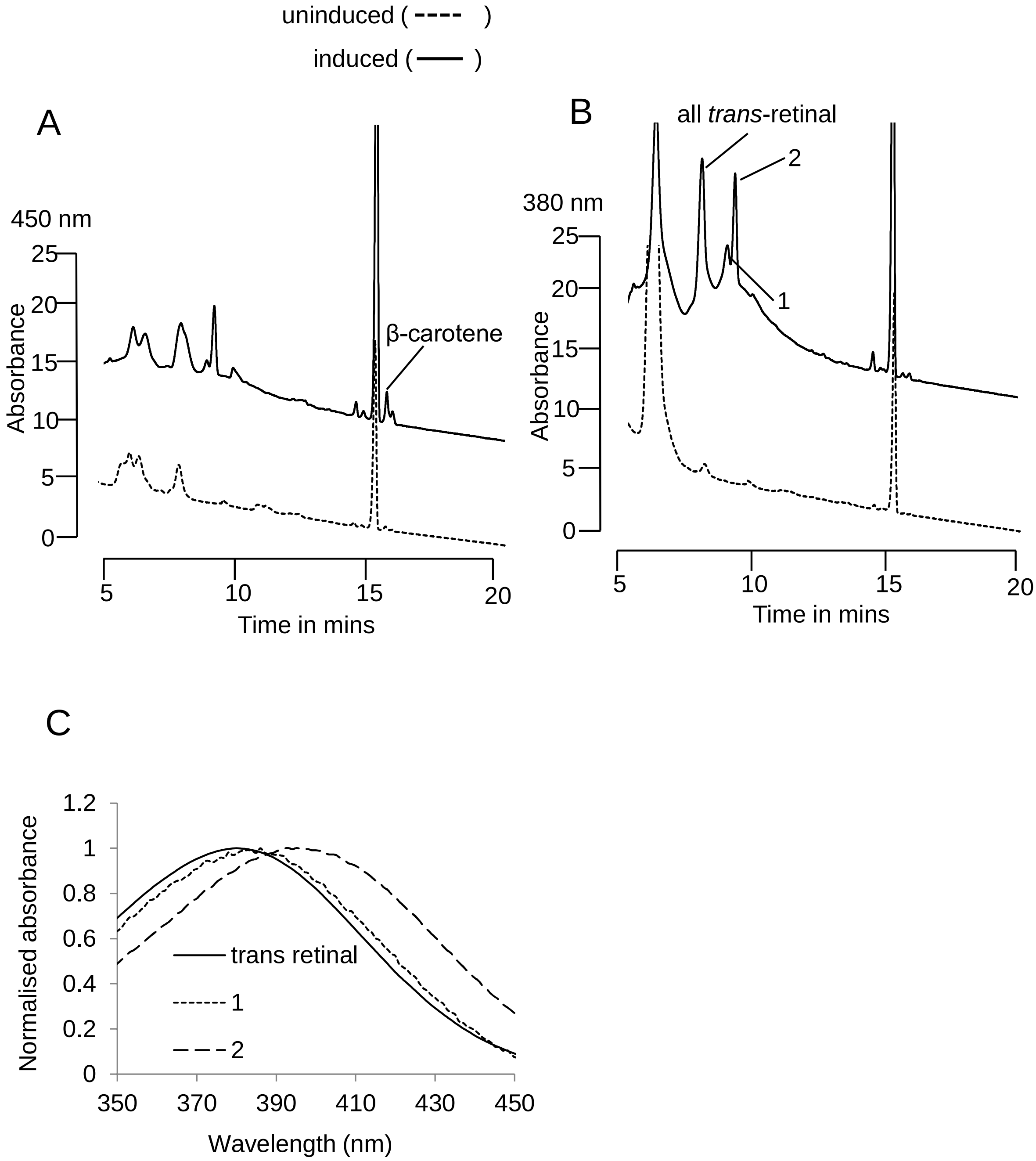

Supplementary figure S3. HPLC elution profiles at 450nm (A) and 380nm (B) of solvent extracted H16Δ*pha* blhDxrCRT-GR uninduced (solid line) and induced (broken line) cell pellets with the β-carotene and retinal peaks shown arrowed; two unknown peaks appearing after induction are labelled 1 and 2 with their normalised absorbance spectra shown compared to that of the *trans*-retinal peak (C).

Suppl Fig S4

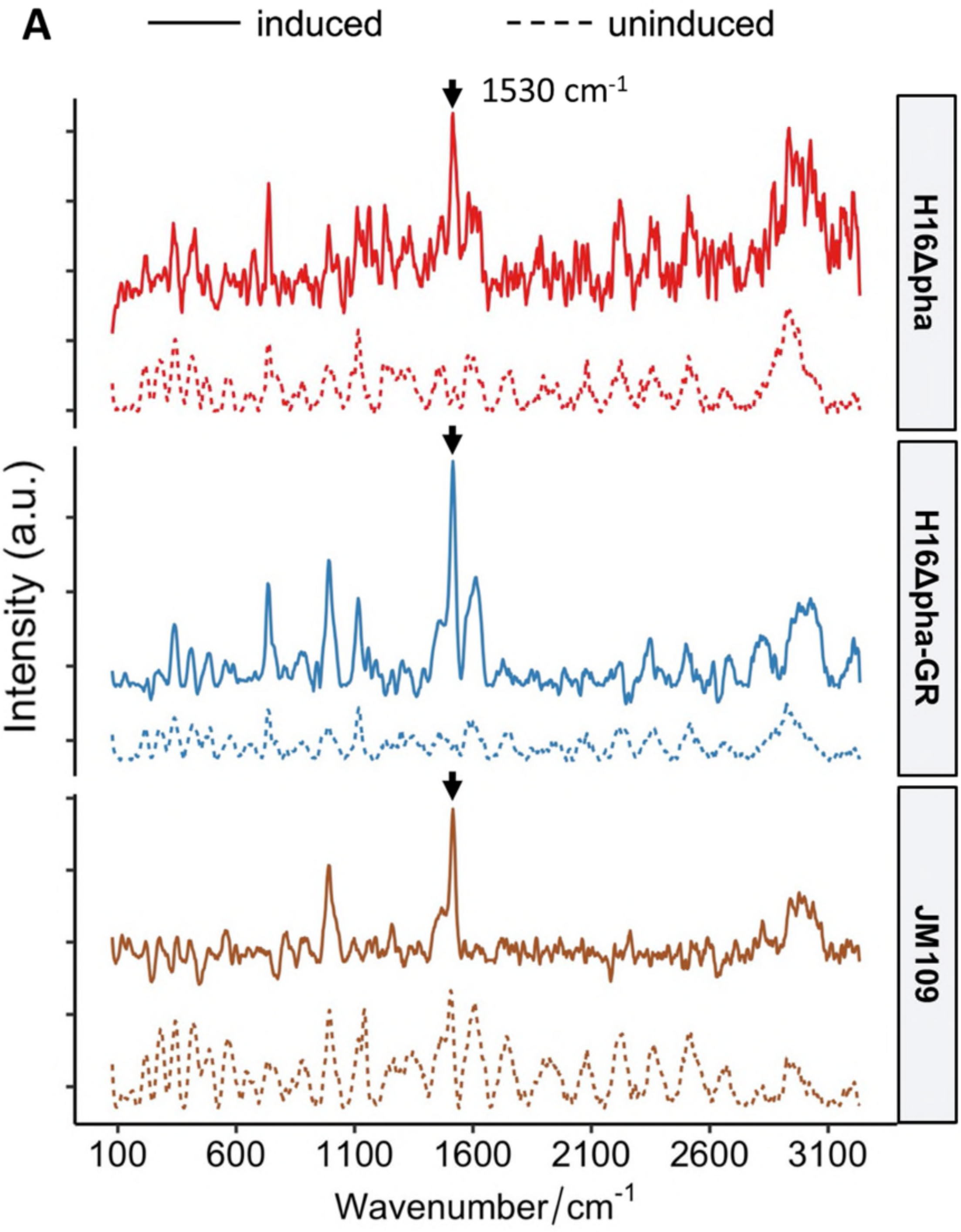
A
induced
uninduced
1530 cm-1
H16Δpha
H16Δpha-GR
JM109
Intensity (a.u.)
100
600
1100
1600
2100
2600
3100
Wavenumber/cm-1

Supp. Fig S4

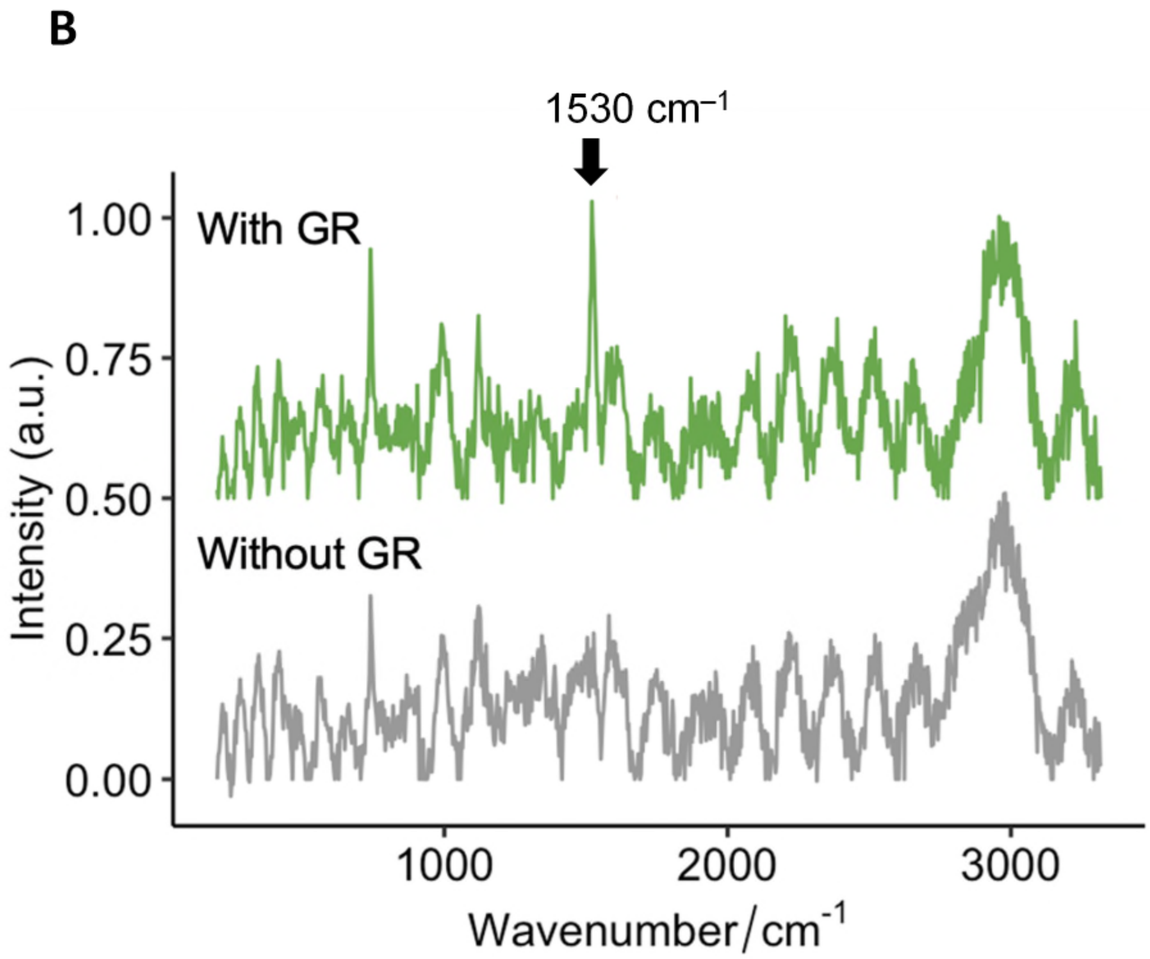

Supplementary figure S4 (A) SCRS of GR in *E. coli* JM109 blhDxrCRT-GR (JM109), *R. eutropha* H16Δ*pha* GR (H16-GR) and *R. eutropha* H16Δ*pha* blhDxrCRT-GR (H16) without induction (broken line) and after induction with arabinose (solid line); strain H16Δ*pha* GR also contained exogenous retinal. (B) SCRS of *R. eutropha* H16 blhDxrCRT-GR without induction (grey trace) and after induction with arabinose (green trace). The Raman signal at 1530 cm^{-1} (arrowed) is indicative of retinal bound within GR.

Suppl Fig S5

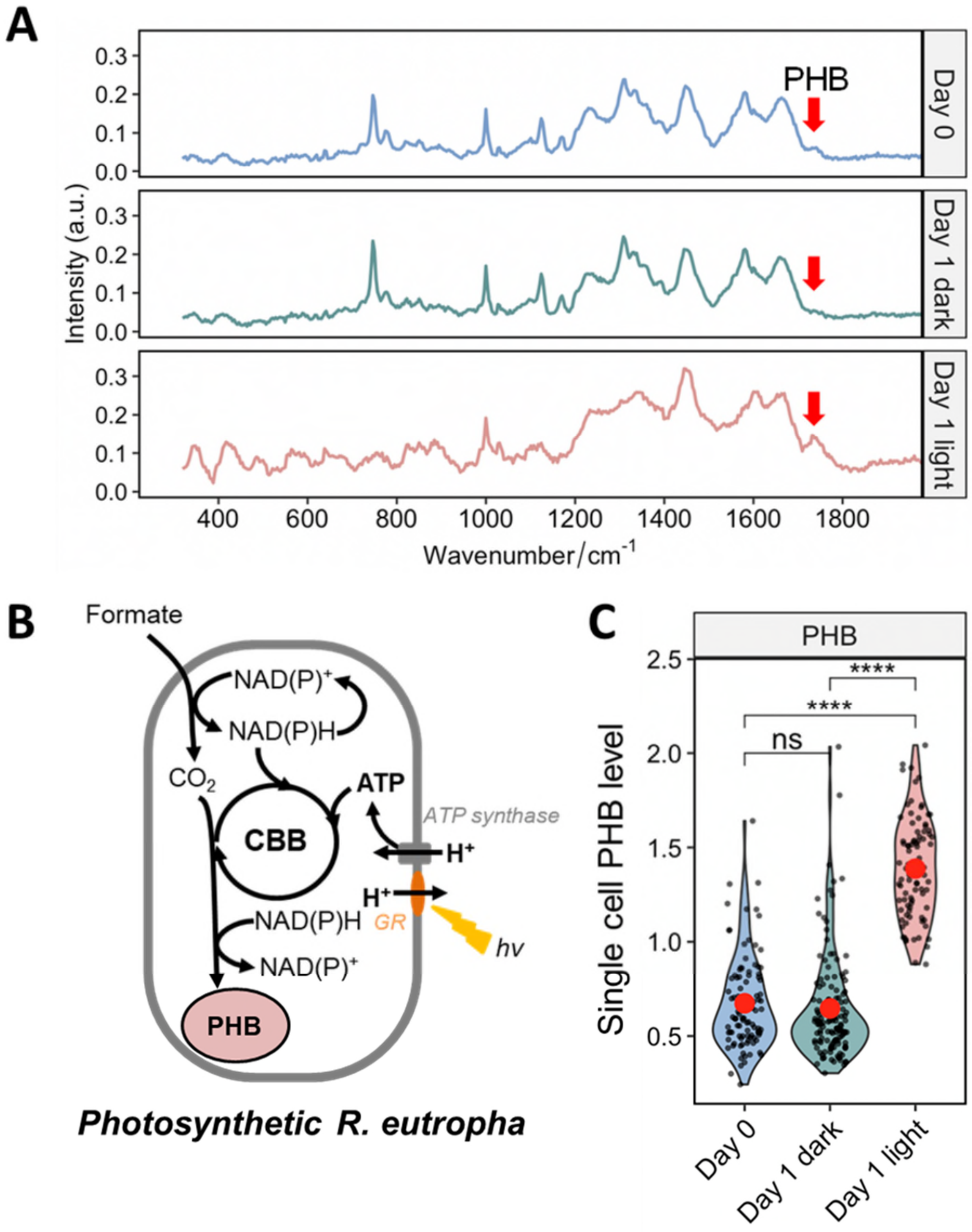

Supplementary figure S5 (A) PHB synthesis from formate by GR-expressing *R. eutropha* H16 (B) Comparisons of single-cell PHB content in the dark and light.

Supp. Fig S6

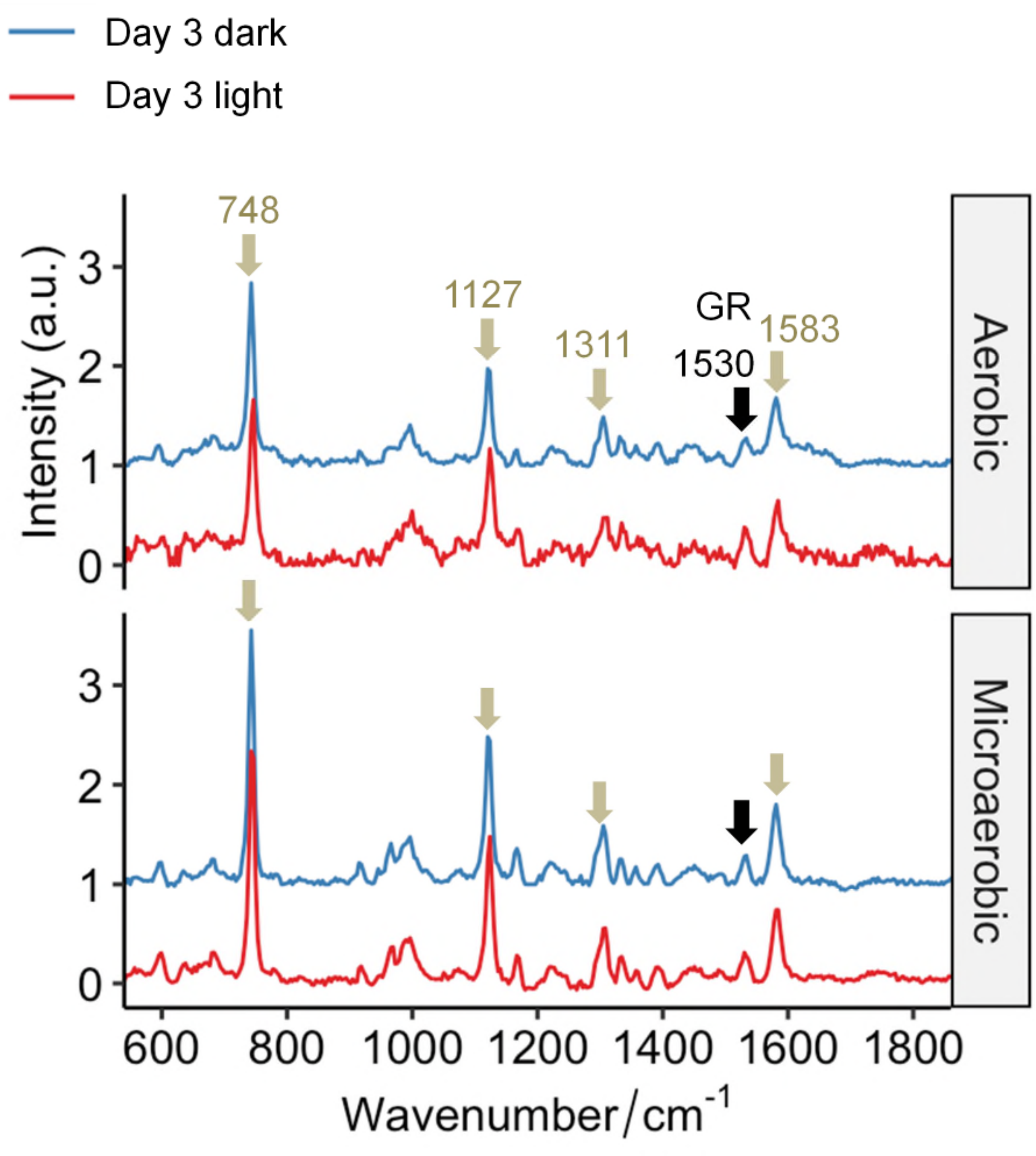

Supplementary figure S6 SCRS of *R. eutropha* H16 Δ*pha* with pLO11a-blhDxrCRT-GR after aerobic and microaerobic growth in MM media containing formate for 72 h under light (red line) or in the dark (blue line). Raman bands representative of the resonant cytochrome *c* molecules are labelled at 1583, 1311, 1123, and 750 cm^{-1}.

Supp. Fig S7

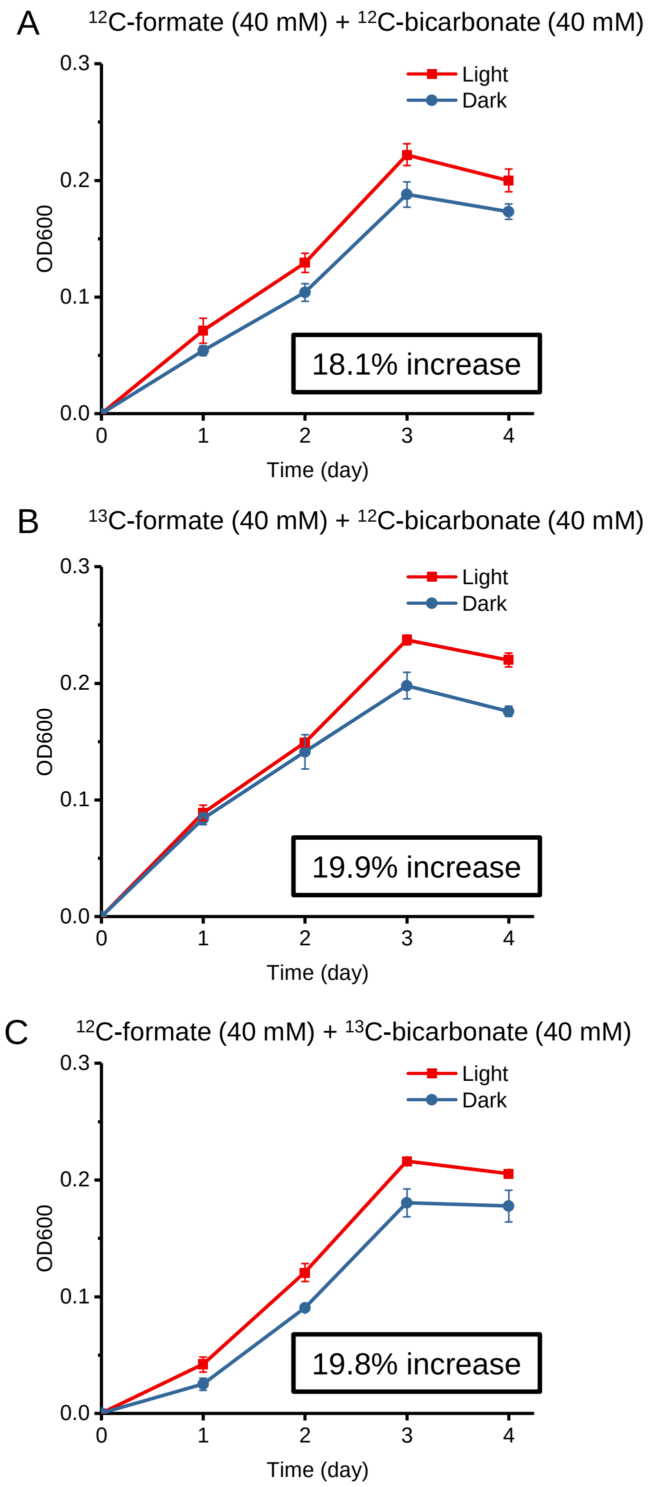

Supplementary figure S7 Growth curves, represented by OD_{600}, of *R. eutropha* H16Δ*pha* with pLO11a-blhDxrCRT-GR grown in (A) ^{12}C-formate + ^{12}C-bicarbonate, (B) ^{13}C-formate +^{12}C-bicarbonate and (C) ^{12}C-formate + ^{13}C-bicarbonate, under microaerobic conditions either in the light or in the dark. The percentage increases under light compared with the dark at the maximum growth point (day 3) are shown for each substrate condition.

Suppl Fig S8

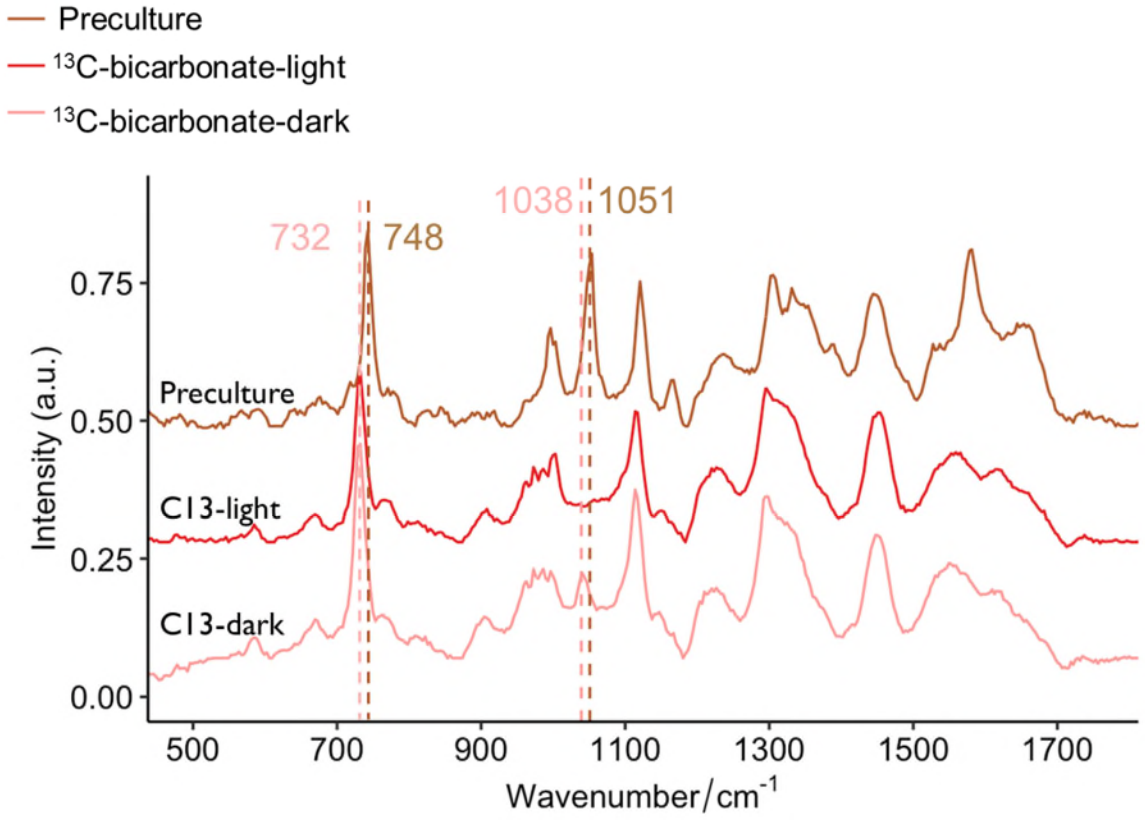

Supplementary figure S8 Single cell Raman spectra of *R. eutropha* H16 with pLO11a-blhDxrCRT-GR at the preculture stage and after 3 days growth with ^{12}C-formate + ^{13}C-bicarbonate under light and dark conditions. The Raman band for cytochrome c was shifted from 748 cm^{-1} to 732 cm^{-1}.

Suppl Fig S9

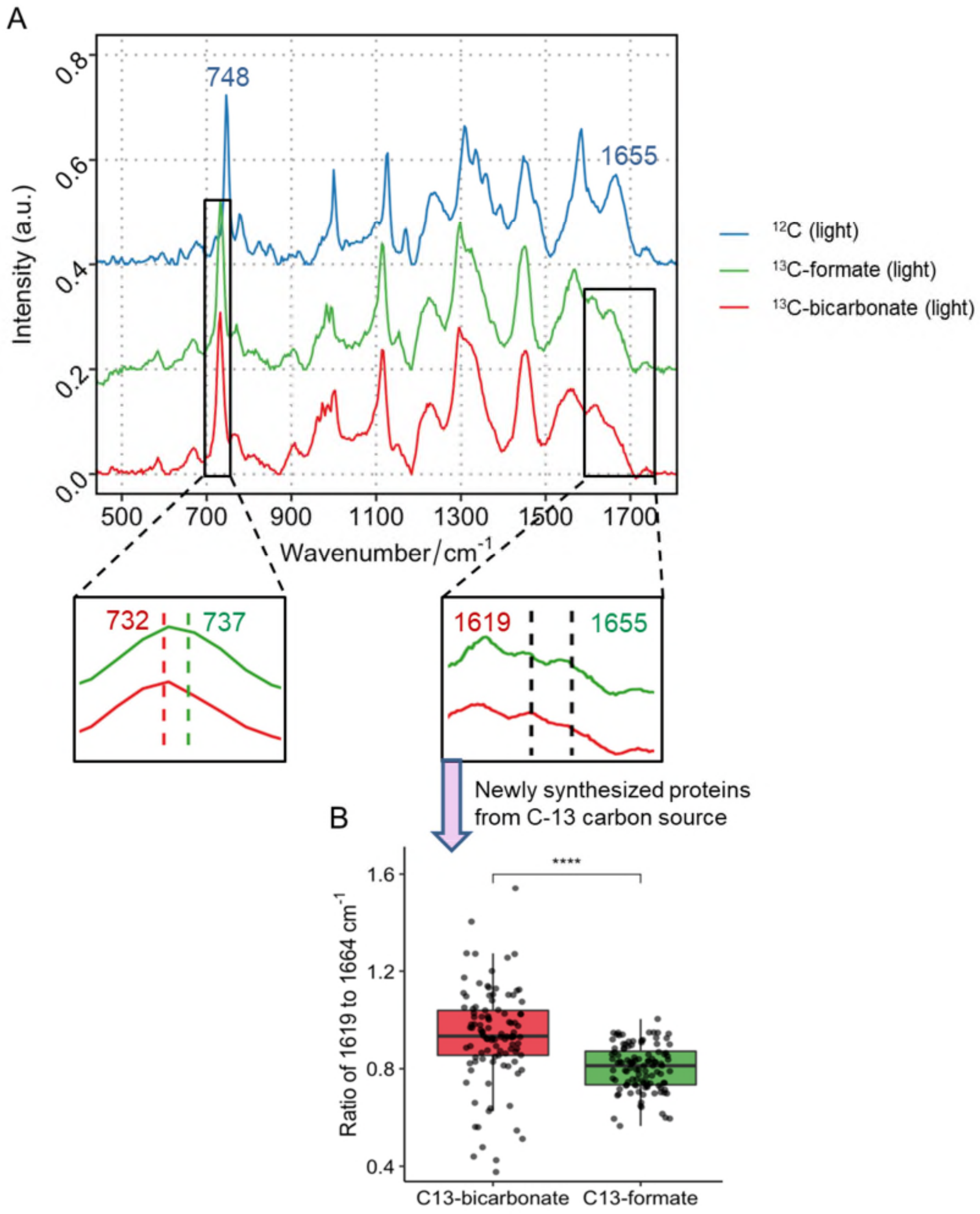

Supplementary figure S9 (A) Single cell Raman spectra of *R. eutropha* H16Δ*pha* with pLO11a-blhDxrCRT-GR grown under light with ^{13}C-formate + ^{12}C-bicarbonate (green) or ^{12}C-formate + ^{13}C-bicarbonate (red). Raman shifts were observed at 737 (^{13}C-formate) and 732 (^{13}C-bicarbonate), and from 1664 to 1619 (both ^{13}C-formate and ^{13}C-bicarbonate). (B) Quantification of newly synthesised proteins at the single-cell level from ^{13}C-formate (green) or ^{13}C-bicarbonate (red) by calculating the ratio of the areas of the 1619-cm^{-1} band to the 1664-cm^{-1} band. Statistical significance was calculated based on Student's t test ($p < 0.0001$).

Suppl Fig S10

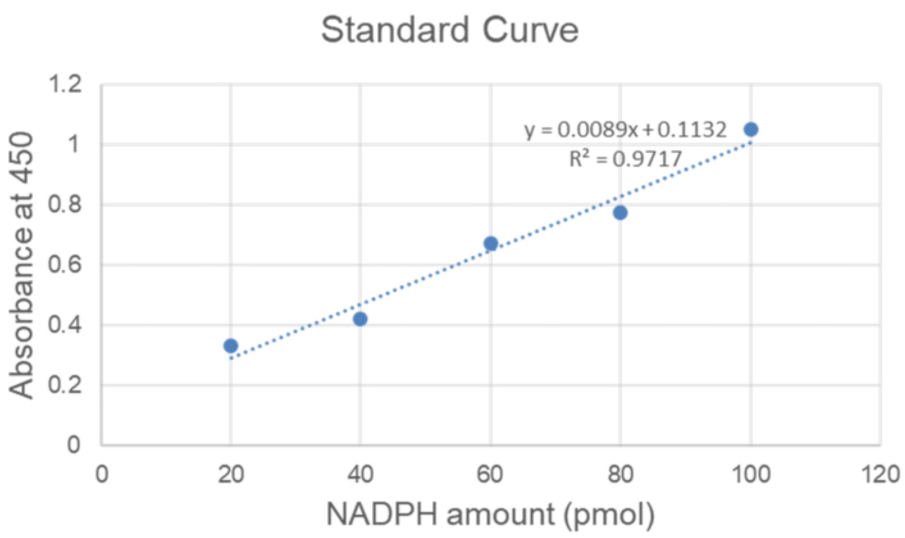

Supplementary figure S10 A standard calibration curve for NADPH based on absorbance at 450 nm using the NADP/NADPH quantification kit (Sigma-Aldrich).

Suppl Fig S11

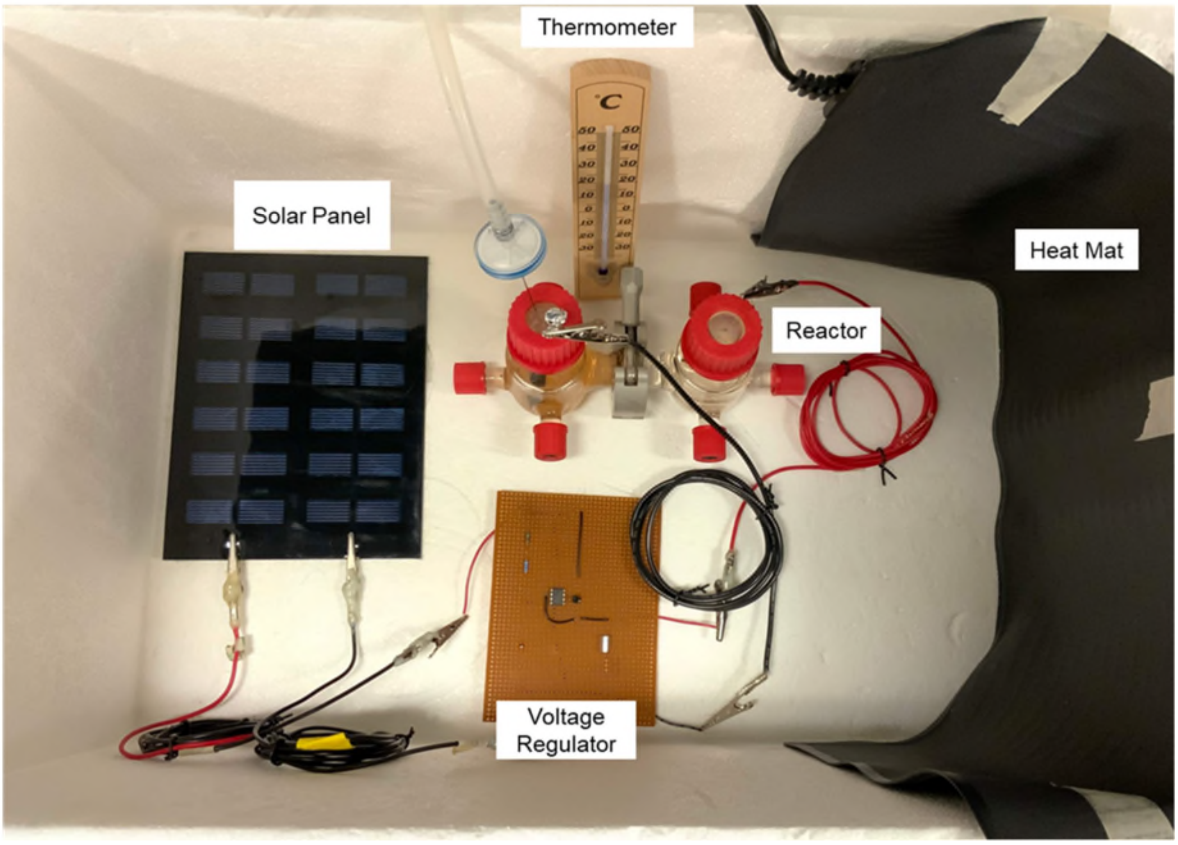

Supplementary figure S11 The setup of the photo-electrosynthetic system.

Suppl Fig S12

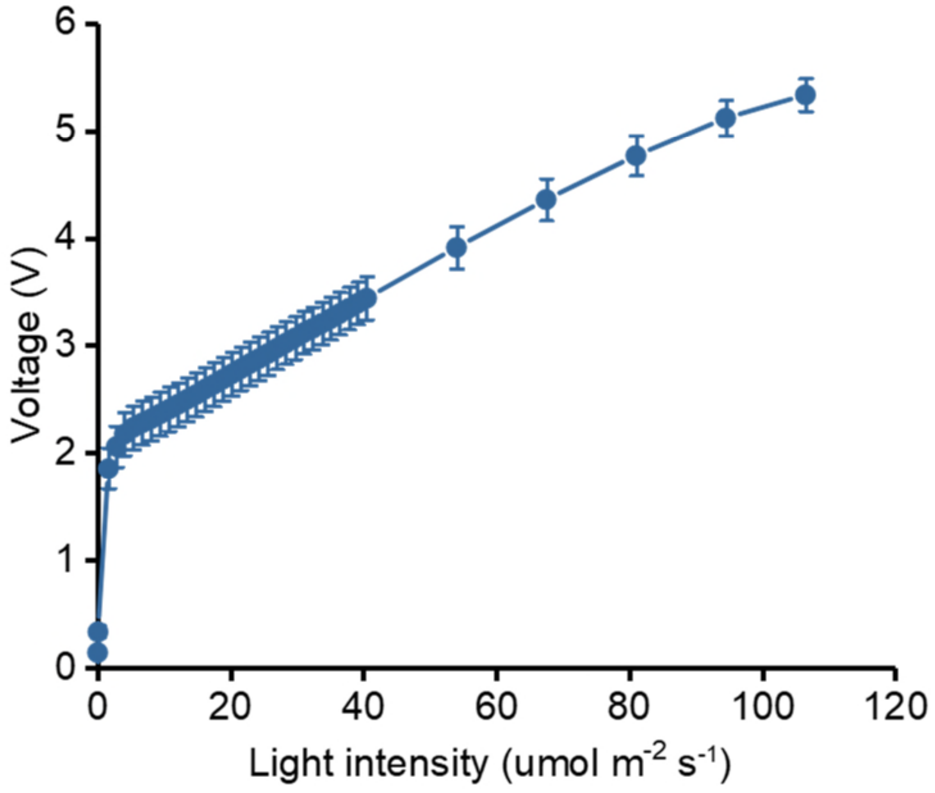

Supplementary figure S12 The electricity production from the solar panel.
